# Sex-specific glioma genome-wide association study identifies new risk locus at 3p21.31 in females, and finds sex-differences in risk at 8q24.21

**DOI:** 10.1038/s41598-018-24580-z

**Published:** 2018-05-09

**Authors:** Quinn T. Ostrom, Ben Kinnersley, Margaret R. Wrensch, Jeanette E. Eckel-Passow, Georgina Armstrong, Terri Rice, Yanwen Chen, John K. Wiencke, Lucie S. McCoy, Helen M. Hansen, Christopher I. Amos, Jonine L. Bernstein, Elizabeth B. Claus, Dora Il’yasova, Christoffer Johansen, Daniel H. Lachance, Rose K. Lai, Ryan T. Merrell, Sara H. Olson, Siegal Sadetzki, Joellen M. Schildkraut, Sanjay Shete, Joshua B. Rubin, Justin D. Lathia, Michael E. Berens, Ulrika Andersson, Preetha Rajaraman, Stephen J. Chanock, Martha S. Linet, Zhaoming Wang, Meredith Yeager, GliomaScan consortium, Laura E. Beane Freeman, Stella Koutros, Demetrius Albanes, Kala Visvanathan, Victoria L. Stevens, Roger Henriksson, Dominique S. Michaud, Maria Feychting, Anders Ahlbom, Graham G. Giles, Roger Milne, Roberta McKean-Cowdin, Loic Le Marchand, Meir Stampfer, Avima M. Ruder, Tania Carreon, Göran Hallmans, Anne Zeleniuch-Jacquotte, J. Michael Gaziano, Howard D. Sesso, Mark P. Purdue, Emily White, Ulrike Peters, Julie Buring, Richard S. Houlston, Robert B. Jenkins, Beatrice Melin, Melissa L. Bondy, Jill. S. Barnholtz-Sloan

**Affiliations:** 1 Department of Medicine, Section of Epidemiology and Population Sciences, Dan L. Duncan Comprehensive Cancer Center, Baylor College of Medicine, Houston, Texas United States of America; 2 Case Comprehensive Cancer Center, Case Western Reserve University School of Medicine, Cleveland, Ohio, United States of America; 3 Department of Population and Quantitative Heath Sciences, Case Western Reserve University School of Medicine, Cleveland, Ohio, United States of America; 4 Division of Genetics and Epidemiology, The Institute of Cancer Research, Sutton, Surrey, United Kingdom; 5 Department of Neurological Surgery and Institute of Human Genetics, School of Medicine, University of California, San Francisco, San Francisco, California, United States of America; 6 Division of Biomedical Statistics and Informatics, Mayo Clinic College of Medicine, Rochester, Minnesota United States of America; 7 Institute for Clinical and Translational Research, Dan L. Duncan Comprehensive Cancer Center, Baylor College of Medicine, Houston, Texas United States of America; 8 Department of Epidemiology and Biostatistics, Memorial Sloan Kettering Cancer Center, New York, New York, United States of America; 9 School of Public Health, Yale University, New Haven, Connecticut United States of America; 10 Department of Neurosurgery, Brigham and Women’s Hospital, Boston, Massachusetts, United States of America; 11 Department of Epidemiology and Biostatistics, School of Public Health, Georgia State University, Atlanta, Georgia United States of America; 12 Cancer Control and Prevention Program, Department of Community and Family Medicine, Duke University Medical Center, Durham, North Carolina United States of America; 13 Duke Cancer Institute, Duke University Medical Center, Durham, North Carolina United States of America; 14 Oncology clinic, Finsen Center, Rigshospitalet, Copenhagen, Denmark; 15 Survivorship Research Unit, The Danish Cancer Society Research Center, Copenhagen, Denmark; 16 Department of Neurology, Mayo Clinic Comprehensive Cancer Center, Mayo Clinic, Rochester, Minnesota United States of America; 17 Department of Neurology, Keck School of Medicine, University of Southern California, Los Angeles, California, United States of America; 18 Department of Preventive Medicine, Keck School of Medicine, University of Southern California, Los Angeles, California, United States of America; 19 Department of Neurology, NorthShore University HealthSystem, Evanston, Illinois United States of America; 20 Cancer and Radiation Epidemiology Unit, Gertner Institute, Chaim Sheba Medical Center, Tel Hashomer, Israel; 21 Department of Epidemiology and Preventive Medicine, School of Public Health, Sackler Faculty of Medicine, Tel-Aviv University, Tel-Aviv, Israel; 22 Department of Public Health Sciences, University of Virginia School of Medicine, Charlottesville, Virginia United States of America; 23 Department of Biostatistics, University of Texas MD Anderson Cancer Center, Houston, Texas United States of America; 24 Department of Pediatrics, Washington University School of Medicine, St. Louis, Missouri United States of America; 25 Department of Neuroscience, Washington University School of Medicine, St. Louis, Missouri United States of America; 26 Department of Stem Cell Biology and Regenerative Medicine, Cleveland Clinic Foundation, Cleveland, Ohio, United States of America; 27 Cancer and Cell Biology Division, The Translational Genomics Research Institute, Phoenix, Arizona United States of America; 28 Department of Radiation Sciences, Faculty of Medicine, Umeå University, Umeå, Sweden; 29 Division of Cancer Epidemiology and Genetics, National Cancer Institute, Rockville, Maryland United States of America; 30 Core Genotyping Facility, National Cancer Institute, SAIC-Frederick, Inc, Gaithersburg, Maryland United States of America; 31 Department of Laboratory Medicine and Pathology, Mayo Clinic Comprehensive Cancer Center, Mayo Clinic, Rochester, Minnesota United States of America; 32 Department of Epidemiology, John Hopkins Bloomberg School of Public Health, Baltimore, Maryland United States of America; 33 American Cancer Society, Atlanta, Georgia United States of America; 34 Department of Oncology, Karolinska University Hospital, Stockholm, Sweden; 35 Department of Public Health and Community Medicine, Tufts University School of Medicine, Boston, Massachusetts, United States of America; 36 Institute of Environmental Medicine, Karolinska Institutet, Stockholm, Sweden; 37 Cancer Epidemiology and Intelligence Division, Cancer Council Victoria, Melbourne, Australia; 38 Department of Public Health, John A. Burns School of Medicine, University of Hawaii at Manoa, Manoma, Hawaii United States of America; 39 Department of Epidemiology, Harvard T.H. Chan School of Public Health, Harvard University, Boston, Massachusetts, United States of America; 40 Department of Epidemiology, Nutrition, Harvard T.H. Chan School of Public Health, Harvard University, Boston, Massachusetts, United States of America; 41 National Institute for Occupational Safety and Health, Centers for Disease Control and Prevention, Atlanta, Georgia, United States of America; 42 Division of Surveillance, Hazard Evaluations, and Field Studies, Centers for Disease Control and Prevention, Atlanta, Georgia, United States of America; 43 Department of Public Health and Clinical Medicine, Faculty of Medicine, Umeå University, Umeå, Sweden; 44 Departments of Population Health and Environmental Medicine, New York University School of Medicine, New York, New York, United States of America; 45 Department of Medicine, Brigham and Women’s Hospital, Boston, Massachusetts, United States of America; 46 Department of Epidemiology, School of Public Health and Community Medicine, University of Washington, Seattle, Washington, United States of America

## Abstract

Incidence of glioma is approximately 50% higher in males. Previous analyses have examined exposures related to sex hormones in women as potential protective factors for these tumors, with inconsistent results. Previous glioma genome-wide association studies (GWAS) have not stratified by sex. Potential sex-specific genetic effects were assessed in autosomal SNPs and sex chromosome variants for all glioma, GBM and non-GBM patients using data from four previous glioma GWAS. Datasets were analyzed using sex-stratified logistic regression models and combined using meta-analysis. There were 4,831 male cases, 5,216 male controls, 3,206 female cases and 5,470 female controls. A significant association was detected at rs11979158 (7p11.2) in males only. Association at rs55705857 (8q24.21) was stronger in females than in males. A large region on 3p21.31 was identified with significant association in females only. The identified differences in effect of risk variants do not fully explain the observed incidence difference in glioma by sex.

## Introduction

Glioma is the most common type of primary malignant brain tumor in the United States (US), with an average annual age-adjusted incidence rate of 6.0/100,000^1^. Glioma can be broadly classified into glioblastoma (GBM, 61.9% of gliomas in adults 18+ in the US) and lower-grade glioma (non-GBM glioma, 24.2% of adult gliomas) with tumors such as ependymoma (6.3%), unclassified malignant gliomas (5.1%), and pilocytic astrocytoma (1.9%) making up the majority of other cases^1^. Many environmental exposures have been investigated as sources of glioma risk, but the only validated risk factors for these tumors are ionizing radiation (which increases risk), and history of allergies or other atopic disease (which decreases risk)^2^. These tumors are significantly more common in people of European ancestry, in males and in older adults^1^. The contribution of common low-penetrance SNPs to the heritability of sporadic glioma in persons with no documented family history is estimated to be ~25%^3^. A recent glioma genome-wide association study (GWAS) meta-analysis validated 12 previously reported risk loci^4^, and identified 13 new risk loci. These 25 loci in total are estimated to account for ~30% of heritable glioma risk. This suggests that there are both undiscovered environmental risk (which accounts for ~75% of incidence variance) and genetic risk factors (accounting for ~70% of heritable risk)^3,4^.

**Figure 1:**
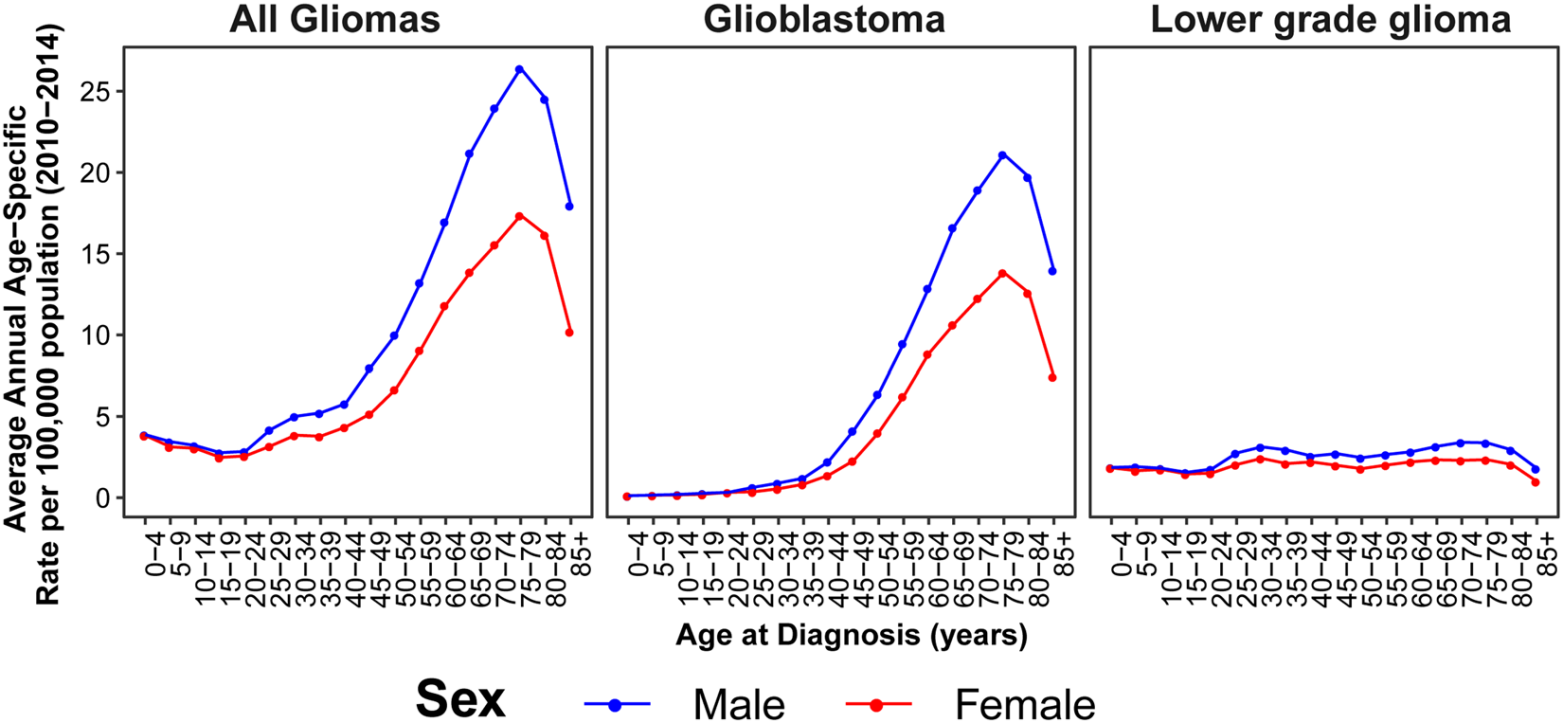
Average Annual Incidence of all glioma, glioblastoma and lower grade glioma by sex and age at diagnosis (CBTRUS 2010–2014).

Population-based studies consistently demonstrate that incidence of gliomas varies significantly by sex. Most glioma histologies occur with a 30–50% higher incidence in males, and this male preponderance of glial tumors increases with age in adult glioma (Fig. 1)^1^. Several studies have attempted to estimate the influence of lifetime estrogen and progestogen exposure on glioma risk in women^5,6^. Results of these analyses have been mixed, and it is not possible to conclusively determine the impact of hormone exposure on glioma risk. Male predominance in incidence occurs broadly across multiple cancer types and is also evident in cancers that occur in pre-pubertal children and in post-menopausal adults^7,8^. Together these observations suggest that other mechanisms in addition to acute sex hormone actions must be identified to account for the magnitude of sex difference in glioma incidence.

Though sex differences exist in glioma incidence, sex differences have not been interrogated in previous glioma GWAS. Sex-specific analyses have the potential to reveal genetic sources of sexual dimorphism in risk, as well as to increase power for detection of loci where effect size or direction may vary by sex^9,10^. The aim of this analysis is to investigate potential sex-specific sources of genetic risk for glioma that may contribute to observed sex-specific incidence differences.

## Results

### Study population

There were 4,831 male cases, 5,216 male controls, 3,206 female cases, and 5,470 female controls (Table 1). A slightly larger proportion of male cases were GBM (58.7% of male cases vs 52.5% of female cases). Controls were slightly older than cases. GBM cases had a higher mean age than non-GBM cases, which was consistent with known incidence patterns of these tumors. Male and female cases within histology groups had similar age at diagnosis. The proportion of non-GBM cases varied by study due to differing recruitment patterns and study objectives (see original publications for details of recruitment patterns and inclusion criteria)^4,11–14^.

**Table 1:** Population characteristics by study and sex.

Characteristic	Study	Males		Females	
		Cases	Controls	Cases	Controls
N	**Total**	**4,831**	**5,216**	**3,206**	**5,470**
	GICC^a^	2,733	1,868	1,831	1,397
	SFAGS-GWAS^b^	440	749	237	1,611
	MDA-GWAS^c^	714	1,094	429	1,142
	GliomaScan^d^	944	1,465	709	1,260
Mean Age (SD)	**Total**	**52**.**5** (**14**.5)**	**58**.**2** (**15**.2)**	**51**.**8** (**14**.9)**	**54**.**7** (**14**.5)**
	GICC	52.5 (14.3)	56.1 (13.4)	51.3 (14.6)	53.4 (14.3)
	SFAGS-GWAS	53.8 (13.0)	50.6 (14.8)	53.5 (14.0)	49.3 (13.2)
	MDA-GWAS	47.1 (13.0)	Modal age group: 60–69^**e**^	47.7 (13.9)	Modal age group: 65–69^**f**^
	GliomaScan	56.0 (15.5)	69.3 (12.7)	55.1 (15.7)	64.0 (15.4)
GBM (% of total)^g^	**Total**	**2**,**835** (**58**.7%)**	**—**	**1**,**682** (**52**.5%)**	**—**
	GICC	1,575 (57.6%)	**—**	885 (48.3%)	**—**
	SFAGS-GWAS	333 (75.7%)	**—**	178 (75.1%)	**—**
	MDA-GWAS	397 (55.6%)	**—**	246 (57.3%)	**—**
	GliomaScan	530 (56.1%)	**—**	373 (52.6%)	**—**
GBM - Mean Age (SD)	**Total**	**57**.**3** (**12**.**0)** **	**—**	**57**.**8** (**12**.**1)** **	**—**
	GICC	57.7 (11.4)	**—**	57.8 (11.6)	**—**
	SFAGS-GWAS	56.4 (11.5)	**—**	56.2 (12.3)	**—**
	MDA-GWAS	52.0 (11.7)	**—**	53.7 (11.3)	**—**
	GliomaScan	60.4 (13.0)	**—**	61.4 (12.5)	**—**
Non-GBM (% of total)^g^	**Total**	**1**,**716** (**35**.5%)**	**—**	**1**,**320** (**41**.2%)**	**—**
	GICC	1,036 (37.9%)	**—**	862 (47.1%)	**—**
	SFAGS-GWAS	107 (24.3%)	**—**	59 (24.9%)	**—**
	MDA-GWAS	317 (44.4%)	**—**	183 (42.7%)	**—**
	GliomaScan	256 (27.1%)	**—**	216 (30.5%)	**—**
Non-GBM - Mean Age (SD)	**Total**	**44**.**3** (**14**.4)**	**—**	**43**.**9** (**14**.3)**	**—**
	GICC	44.7 (14.6)	**—**	44.6 (14.2)	**—**
	SFAGS-GWAS	45.7 (14.2)	**—**	45.4 (15.8)	**—**
	MDA-GWAS	41.0 (11.9)	**—**	39.6 (12.9)	**—**
	GliomaScan	46.3 (15.5)	**—**	44.4 (15.2)	**—**

^a^Data from Glioma International Case-Control Study (GICC; Melin, *et al*.^4^); ^b^Data from San Francisco Adult Glioma Study GWAS (SFAGS-GWAS; Wrensch, *et al*.^12^); ^c^data from MD Anderson Cancer Center GWAS (MDA-GWAS; Shete, *et al*.^13^); ^d^Data from the National Cancer Institute’s GliomaScan (GliomaScan; Rajaraman, *et al*.^14^); ^e^Data from CGEMS prostate study (Yeager *et al*.^35^). Continuous age is not available, age distribution is as follows 50–59: 12.3%, 60–69: 56.7%, 70–79: 30.7%, 80–89: 0.3%; ^f^Data from CGEMS breast study (Hunter *et al*.^36^). Continuous age is not available, age distribution is as follows: 0–54: 4.3%, 55–59: 15.0%, 60–64: 23.6%, 65–69: 27.5%, 70–74: 19.0%, 75–99: 10.7%; ^g^Histology information not available for all cases and frequencies may not add to 100%.

**Differs between included studies at the p < 0.05 level.

### Previously discovered glioma risk regions

**Figure 2:**
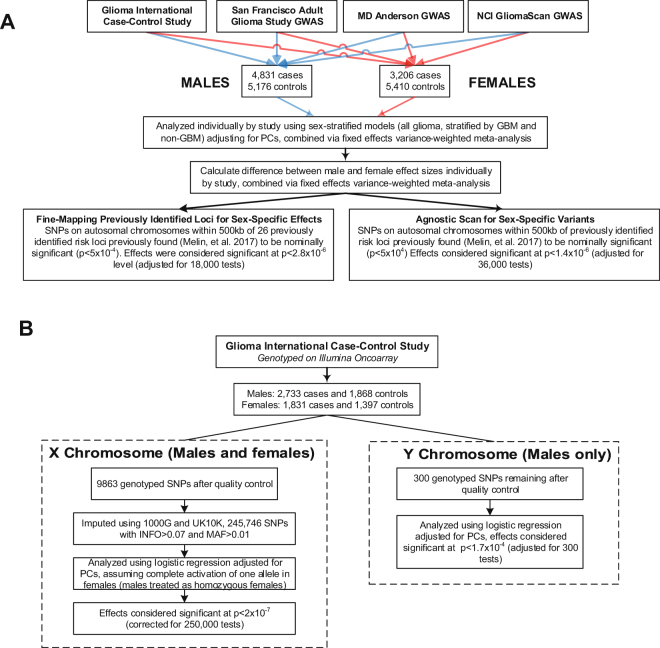
Study Schematic for analyses of (**A**) autosomal SNPs and (**B**) SNPs on sex chromosomes.

There were 5,934 SNPs within 500 kb of 26 previously discovered glioma risk loci with IMPUTE2 information score (INFO) > 0.7 and MAF > 0.01 that were previously found to have at least a nominal (p < 5 × 10^−4^) association with glioma^4^, and results were considered significant at p < 2.8 × 10^−6^ level (adjusted for 6,000 tests in each of three histologies [18,000 tests], see Fig. 2A for schematic of study design). Among the 25 previously validated glioma risk loci, nine loci contained 10 SNPs with p_M_ < 2.8 × 10^−6^ and/or p_F_ < 2.8 × 10^−6^ in any histology: 1p31.3 (*RAVER*2), 5p15.33 (*TERT*), 7p11.2 (*EGFR*, two independent loci), 8q24.21 (intergenic region near *MYC*), 9p21.3 (*CDKN2B*-*AS1*), 11q23.3 (*PHLDB1*), 16p13.3 (*RHBDF1*), 17p13.1 (*TP53*), and 20q13.33 (*RTEL1*) (Table 2). OR_M_ and OR_F_ were similar in the majority of these loci.

**Table 2:** Previously identified glioma risk loci and histology-specific odds ratios (OR) and 95% confidence intervals (95% CI) stratified by sex.

SNP (Locus)	Risk Allele	Histology	Males		Females		P_D_
			P_M_	OR_M_ (95% CI)	P_F_	OR_F_ (95% CI)	
rs12752552 (1p31.3)	**T**/C	All glioma	1.40 × 10^−6^	1.25 (1.14–1.37)	3.22 × 10^−4^	1.21 (1.09–1.34)	0.7280
		GBM	3.27 × 10^−6^	1.28 (1.15–1.42)	8.41 × 10^−4^	1.24 (1.09–1.41)	0.7535
		Non-GBM	0.0235	1.15 (1.02–1.30)	0.0036	1.23 (1.07–1.42)	0.4252
rs9841110 (3p21.31)	**C** **/G**	All glioma	0.5885	1.02 (0.96–1.08)	5.55 × 10^–8^	1.22 (1.14–1.32)	1.77 × 10^–4^
		GBM	0.3429	1.04 (0.96–1.11)	1.44 × 10^−7^	1.27 (1.16–1.38)	6.04 × 10^−4^
		Non-GBM	0.4816	0.97 (0.89–1.06)	0.0160	1.13 (1.02–1.24)	0.0186
rs10069690 (5p15.33)	C/**T**	All glioma	7.58 × 10^−31^	1.49 (1.39–1.60)	4.88 × 10^−20^	1.45 (1.34–1.57)	0.5688
		GBM	3.38 × 10^−35^	1.64 (1.52–1.78)	6.29 × 10^–22^	1.60 (1.45–1.76)	0.7049
		Non-GBM	1.20 × 10^−6^	1.27 (1.15–1.40)	1.67 × 10^−6^	1.31 (1.17–1.46)	0.7036
rs75061358 (7p11.2)	T/**G**	All glioma	6.93 × 10^−12^	1.43 (1.29–1.59)	1.71 × 10^−9^	1.46 (1.29–1.66)	0.8114
		GBM	2.66 × 10^−16^	1.65 (1.46–1.86)	1.16 × 10^−11^	1.68 (1.45–1.96)	0.8211
		Non-GBM	0.0079	1.23 (1.06–1.43)	0.0129	1.25 (1.05–1.49)	0.9246
rs11979158 (7p11.2)	**A**/G	All glioma	4.87 × 10^−12^	1.33 (1.23–1.44)	0.0187	1.12 (1.02–1.22)	0.0055
		GBM	1.26 × 10^−12^	1.40 (1.28–1.54)	1.33 × 10^−4^	1.24 (1.11–1.39)	0.1184
		Non-GBM	2.74 × 10^−5^	1.27 (1.13–1.41)	0.9014	0.99 (0.88–1.12)	0.0034
rs55705857 (8q24.21)	A/**G**	All glioma	1.09 × 10^−14^	1.56 (1.40–1.75)	1.22 × 10^−39^	2.45 (2.14–2.80)	3.46 × 10^−7^
		GBM	0.0344	1.17 (1.01–1.34)	4.16 × 10^−7^	1.61 (1.34–1.94)	0.0066
		Non-GBM	8.13 × 10^−36^	2.66 (2.28–3.10)	1.85 × 10^−65^	4.71 (3.94–5.63)	8.44 × 10^−7^
rs634537 (9p21.3)	T/**G**	All glioma	2.37 × 10^−21^	1.33 (1.25–1.41)	6.38 × 10^−14^	1.30 (1.21–1.39)	0.6496
		GBM	1.00 × 10^−20^	1.38 (1.29–1.48)	1.92 × 10^−16^	1.41 (1.30–1.53)	0.6544
		Non-GBM	2.63 × 10^−8^	1.26 (1.16–1.37)	4.88 × 10^−4^	1.18 (1.08–1.30)	0.3131
rs12803321 (11q23.3)	**G**/C	All glioma	3.96 × 10^−4^	1.12 (1.05–1.19)	8.49 × 10^−6^	1.18 (1.10–1.26)	0.2680
		GBM	0.4497	0.97 (0.91–1.04)	0.6463	1.02 (0.94–1.11)	0.3667
		Non-GBM	1.82 × 10^−14^	1.41 (1.29–1.53)	8.88 × 10^−13^	1.43 (1.30–1.57)	0.7207
rs3751667 (16p13.3)	C/**T**	All glioma	2.98 × 10^−6^	1.18 (1.10–1.26)	0.0297	1.09 (1.01–1.19)	0.1779
		GBM	2.22 × 10^−4^	1.16 (1.07–1.26)	0.1130	1.08 (0.98–1.19)	0.2729
		Non-GBM	2.62 × 10^−6^	1.26 (1.14–1.38)	0.0060	1.17 (1.05–1.31)	0.3241
rs78378222 (17p13.1)	T/**G**	All glioma	3.36 × 10^−17^	2.41 (1.97–2.96)	1.75 × 10^−12^	2.43 (1.90–3.12)	0.8483
		GBM	1.27 × 10^−14^	2.65 (2.07–3.40)	2.28 × 10^−9^	2.67 (1.93–3.68)	0.8731
		Non-GBM	1.10 × 10^−10^	2.79 (2.04–3.80)	4.40 × 10^−8^	2.70 (1.89–3.85)	0.9385
rs2297440 (20q13.33)	T/**C**	All glioma	4.09 × 10^−21^	1.42 (1.32–1.52)	1.34 × 10^−13^	1.37 (1.26–1.49)	0.5299
		GBM	1.22 × 10^−19^	1.47 (1.35–1.59)	1.15 × 10^−16^	1.53 (1.39–1.70)	0.5159
		Non-GBM	2.92 × 10^−7^	1.29 (1.17–1.43)	0.0040	1.18 (1.05–1.32)	0.1916

**Figure 3:**
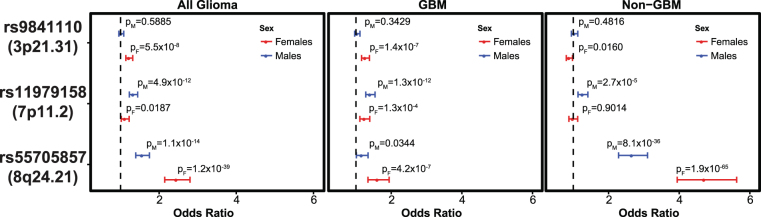
Sex-specific odds ratios overall and by histology grouping, 95% CI and p values for selected previous GWAS hits and 3p21.31 (rs9841110) for all glioma, GBM, and non-GBM.

For one of two independent loci at 7p11.2 (rs11979158), there was a significant association only in males for all glioma (OR_M_ = 1.33 [95% CI = 1.23–1.44], p_M_ = 4.87 × 10^−12^) and GBM (OR_M_ = 1.40 [95% CI = 1.28–1.54], p_M_ = 1.26 × 10^−12)^ but the sex differences did not meet the significance threshold (overall p_D_ = 0.0055, and GBM p_D_ = 0.1184) (Fig. 3, Table 2).

The previously identified SNP at 8q24.21 (rs55705857) was the most significant SNP in both males and females. Odds ratio for rs55705857 in all glioma was significantly higher in females (OR_F_ = 2.45 [95% CI = 2.14–2.80], p_F_ = 1.22 × 10^−39^) as compared to males (OR_M_ = 1.56 [95% CI = 1.40–1.75], p_M_ = 1.09 × 10^−14^) with p_D_ = 3.46 × 10^−7^. In non-GBM only, OR_F_ (OR_F_ = 4.71 [95% CI = 3.94–5.63], p_F_ = 1.85 × 10^−65^) was also elevated as compared to OR_M_ (OR_M_ = 2.66 [95% CI = 2.28–3.10)], p_M_ = 8.13 × 10^−36^) with p_D_ = 8.44 × 10^−7^ (Fig. 3, Table 2). This association was further explored in a case-only analysis, where there was a significant difference between males and females overall (p = 0.0012), and in non-GBM (p = 0.0084) (Supplemental Table 1).

Previous studies have found a strong association between rs55705857 and oligodendroglial tumors (particularly tumors with isocitrate dehydrogenase 1/2 (*IDH1/2*) mutation and loss of the short arm of chromosome 1 [1p] and the long arm of chromosome 19 [19q]), so this association was further explored in the non-GBM (lower grade glioma [LGG]) histology groups (Table 3**)**. For World Health Organization (WHO) grade II-grade III astrocytoma, effect was stronger in females (OR_F_ = 4.64 [95% CI = 3.53–6.09], p_F_ = 2.15 × 10^−28^) as compared to males (OR_M_ = 2.87 [95% CI = 2.31–3.56], p_M_ = 1.19 × 10^−21^) with p_D_ = 0.0065. For WHO grade II–III oligodendrogliomas effect was stronger than observed in WHO grade II–III astrocytomas, and effect size was stronger in females (OR_F_ = 12.15 [95% CI = 8.96–16.48], p_F_ = 3.68 × 10^−58^) as compared to males (OR_M_ = 5.47 [95% CI = 4.16–7.19], p_M_ = 5.37 × 10^−34^) with p_D_ = 6.60 × 10^−5^. Oligoastrocytic tumors were not included in sub-analyses due to recent research that suggests that these tumors are not an entity that is molecularly distinct from oligodendrogliomas or astrocytomas^15^.

**Table 3:** Sex-specific odds ratios (OR), 95% confidence intervals (95% CI), and p values from meta-analysis for rs11979158, rs55705857 and rs9841110 by specific non-GBM histologies.

RSID (Locus)	Histology	Males			Females			P_D_
		P_M_	OR_M_ (95% CI)	P_het_	P_F_	OR_F_ (95% CI)	P_het_	
rs9841110 (3p21.31)	Astrocytoma (Non-GBM) (WHO grade II-III)	0.5304	1.04 (0.92–1.17)	0.751	0.0407	1.15 (1.01–1.32)	0.549	0.2409
	Oligodendroglioma (WHO grade II-III)	0.4190	0.94 (0.81–1.09)	0.694	0.0973	1.14 (0.98–1.34)	0.360	0.0649
rs11979158 (7p11.2)	Astrocytoma (Non-GBM) (WHO grade II-III)	0.0023	0.79 (0.68–0.92)	0.056	0.9363	0.99 (0.83–1.18)	0.418	0.0500
	Oligodendroglioma (WHO grade II-III)	0.0221	0.81 (0.68–0.97)	0.865	0.6561	1.05 (0.86–1.28)	0.262	0.0471
rs55705857 (8q24.21)	Astrocytoma (Non-GBM) (WHO grade II-III)	1.19 × 10^−21^	2.87 (2.31–3.56)	0.073	2.15 × 10^−28^	4.64 (3.53–6.09)	0.237	0.0065
	Oligodendroglioma (WHO grade II-III)	5.37 × 10^−34^	5.47 (4.16–7.19)	0.103	3.68 × 10^−58^	12.15 (8.96–16.48)	0.027	6.60 × 10^−5^

### Genome-wide scan of nominally significant regions

**Figure 4:**
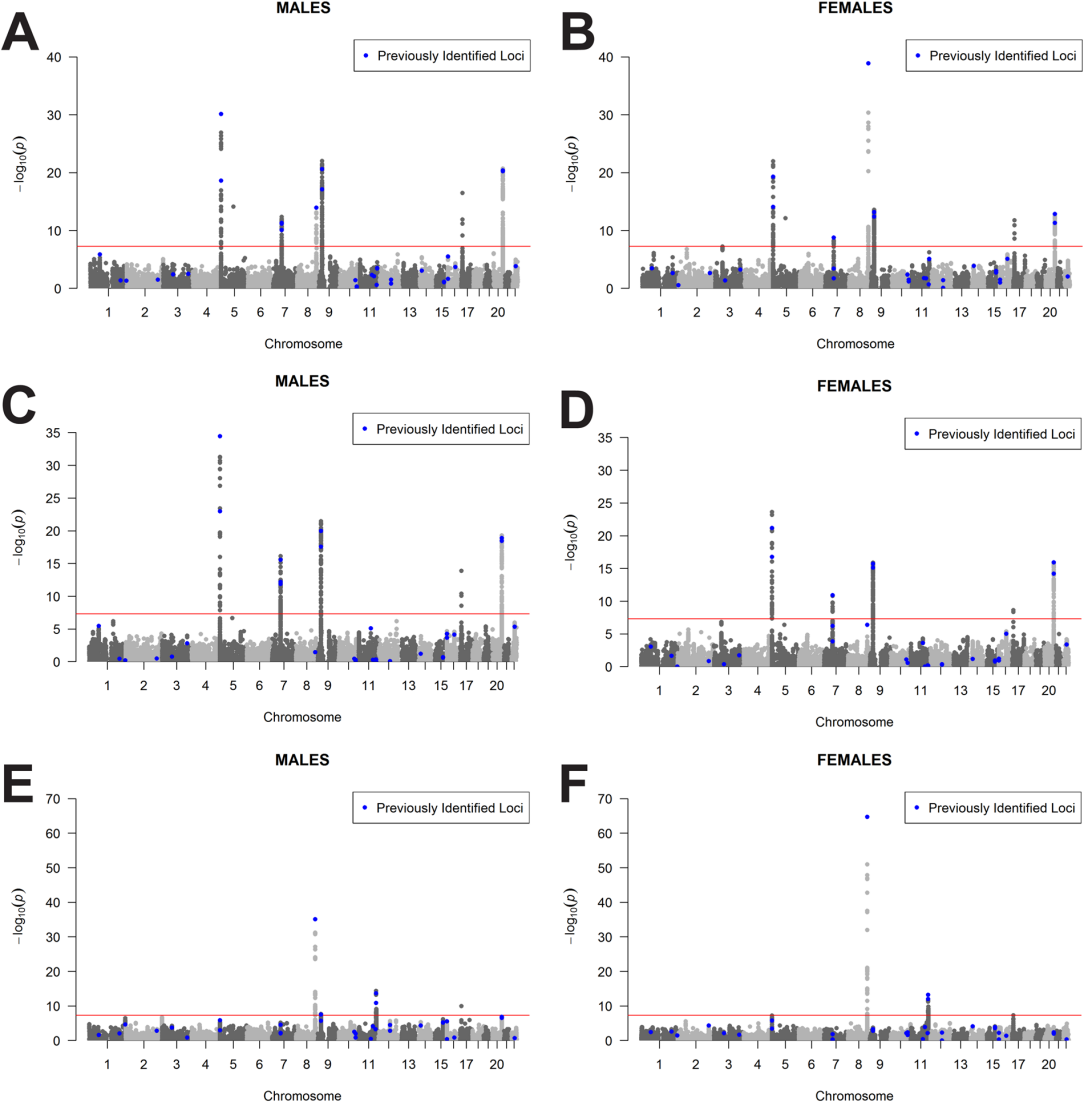
Manhattan plot of -log(p) values for all glioma in (**A**) males and (**B**) females, for GBM in (**C**) males and (**D**) females, and for non-GBM in (**E**) males and (**F**) females.

**Figure 5:**
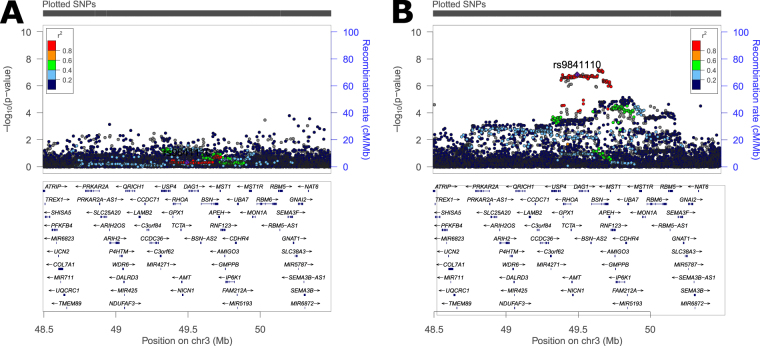
Plot of region on chromosome 3 identified as having a sex-specific association with GBM for (**A**) males and (**B**) females.

In a previous eight study meta-analysis, ~12,000 SNPs (INFO > 0.7, MAF > 0.01) were identified as having a nominally significant (p < 5 × 10^−4^) association with all glioma, GBM, or non-GBM^4^. A sex-stratified genome-wide scan was conducted within this set of SNPs and results were considered significant at p_D_ < 1.4 × 10^−6^ (adjusted for 12,000 tests in each of three histologies [36,000 tests], see Fig. 2A for schematic of study design). Similar genome-wide peaks were observed between males and females (Fig. 4). One large region within 3p21.31 (49400kb–49600kb, ~200 kb) was identified as being significantly associated with glioma and GBM in females only (Fig. 5, Supplemental Fig. 1). There were 243 SNPs with nominally significant associations within this region in the previous eight-study meta-analysis (p < 5 × 10^−4^), and 32 of these had nominally significant sex associations (p_F_ < 5 × 10^−6^ or p_M_ < 5 × 10^−6^) in all glioma or GBM. The strongest association in females within this region was at rs9841110, in both all glioma (OR_F_ = 1.22 [95% CI = 1.14–1.32], p_F_ = 5.55 × 10^−8^) with p_D_ = 1.77 × 10^−4^) and GBM only (OR_F_ = 1.27 [95% CI = 1.16–1.38], p_F_ = 3.86 × 10^−7^) with p_D_ = 6.04 × 10^−4^), while there were no significant associations detected in males (Fig. 3). No SNPs in this region were significantly associated with non-GBM. In a case-only analysis a marginally significant difference was detected between males and females overall (p = 0.0520) and in GBM (p = 0.0428) (Supplemental Table 1).

### Agnostic scan of sex chromosome loci

SNPs on the sex chromosomes were analyzed in GICC only. There were 245,746 SNPs with INFO > 0.7 and MAF > 0.01 on the X chromosome after quality control and imputation, and results were considered significant at p < 2 × 10^−7^ (corrected for 250,000 tests, see Fig. 2B for a schematic of study design). No SNPs met this significance threshold. After quality control procedures were complete, there were 300 SNPs remaining on the Y chromosome. There was no imputation performed of the Y chromosome data and only the 300 genotyped SNPs were evaluated. No significant signals were detected on the Y chromosome.

### Combined analysis of germline variants and somatic characterization

Due to the lack of molecular classification data included in the GICC, MDA-GWAS, SFAGS-GWAS and GliomaScan datasets, glioma data obtained from TCGA datasets (GBM and LGG) were used to explore the potential confounding due to molecular subtype variation with histologies. There were 758 individuals from the TCGA dataset available for analysis with available germline genotyping, molecular characterization, sex, and age data (Supplemental Table 2). Overall, slightly more females (53.2%) as compared to males (47.2%) had *IDH1/2* mutant glioma, but this difference was not statistically significant (p = 0.1104) (Supplemental Fig. 2). When tumors were stratified by histological type, approximately equal proportions of males and females had *IDH1/2* mutations present in their tumors (GBM: 6.0% in males, and 5.2% in females; LGG: 17.9% in males, and 17.7% in females). There were also no significant differences by sex in *IDH*/*TERT*/1p19q subtype (Supplemental Fig. 3, overall p = 0.2859), or pan-glioma methylation subgroup (Supplemental Fig. 4, overall p = 0.4153).

SNPs found to be nominally significant (p < 5 × 10^−4^) in a previous 8 study meta-analysis, with imputation quality (r^2^) ≥ 0.7 were identified within the TCGA germline genotype data and D’ and r^2^ values in CEU were used to select proxy SNPs (Supplemental Table 3)^16^. A case-only analysis was conducted using sex as a binary phenotype for proxy SNPs in the TCGA dataset. In the overall meta-analysis, there was a nominally significant signal in the case-only meta-analysis for the proxy SNP in 3p21.31 in glioblastoma (Table 4). There was no significant association in the TCGA set, but RAF was elevated in females as compared to males in the GBM set, as well as in all *IDH1/2* wild type gliomas. MAF in LGG and *IDH1/2* mutant glioma was similar among males and females. There was a nominally significant signal in the case-only meta-analysis for the proxy SNP at 7p11.2, but no significant association in the TCGA, but RAF was elevated in males as compared to females in the GBM set, as well as in all *IDH1/2* wild type gliomas. There was no significant signal detected in the overall case-only meta-analysis for the proxy SNP at 8q24.21, or within the TCGA set. Among both LGG and *IDH1/2* mutant, RAF was elevated in females as opposed to males.

**Table 4:** Risk allele frequencies (RAF) Case-only odds ratios, 95% confidence intervals (95% CI), and p values for marker SNPs from four study meta-analysis and the Cancer Genome Atlas genotyping data.

Marker SNP	Histology	Four-study Meta-Analysis				The Cancer Genome Atlas				
		Males	Females	Case-only analysis (males:females)		INFO	Males	Females	Case-only analysis (males:females)	
		RAF_cases_	RAF_cases_	P	OR (95% CI)		RAF_cases_	RAF_cases_	p	OR (95% CI)
rs9814873 (3p21.31)	All glioma	0.692	0.707	0.0577	1.07 (1.00–1.15)	1.00	0.701	0.716	0.5321	0.93 (0.75–1.16)
	GBM	0.694	0.716	0.0371	1.11 (1.01–1.22)	1.00	0.697	0.742	0.2003	0.80 (0.58–1.12)
	LGG (non-GBM)	0.686	0.691	0.6446	1.03 (0.92–1.15)	1.00	0.705	0.697	0.8039	1.04 (0.77–1.40)
	*IDH1/2* wild type	**—**	**—**	**—**	**—**	1.00	0.704	0.731	0.4343	0.88 (0.64–1.21)
	*IDH1/2* mutant	**—**	**—**	**—**	**—**	1.00	0.705	0.692	0.7023	1.06 (0.77–1.47)
rs7785013 (7p11.2)	All glioma	0.864	0.847	0.0058	0.88 (0.80–0.96)	0.99	0.855	0.850	0.7813	1.04 (0.78–1.39)
	GBM	0.872	0.861	0.2141	0.92 (0.81–1.05)	0.99	0.854	0.840	0.6073	1.12 (0.73–1.72)
	LGG (non-GBM)	0.855	0.832	0.0109	0.83 (0.72–0.96)	0.99	0.856	0.857	0.9585	0.99 (0.67–1.47)
	*IDH1/2* wild type	**—**	**—**	**—**	**—**	0.99	0.864	0.837	0.3132	1.24 (0.82–1.87)
	*IDH1/2* mutant	**—**	**—**	**—**	**—**	0.99	0.846	0.875	0.2447	0.77 (0.50–1.19)
rs4636162 (8q24.21)	All glioma	0.358	0.365	0.5161	1.02 (0.96–1.09)	0.93	0.392	0.424	0.2113	0.88 (0.71–1.08)
	GBM	0.343	0.338	0.6001	0.98 (0.89–.07)	0.93	0.374	0.404	0.4456	0.89 (0.66–1.20)
	LGG	0.383	0.401	0.1594	1.08 (0.97–1.20)	0.94	0.410	0.438	0.3891	0.88 (0.67–1.17)
	*IDH1/2* wild type	**—**	**—**	**—**	**—**	0.92	0.371	0.373	0.9480	0.99 (0.73–1.34)
	*IDH1/2* mutant	**—**	**—**	**—**	**—**	0.94	0.419	0.460	0.2613	0.84 (0.63–1.14)

### Sex-stratified genotypic risk scores

**Figure 6:**
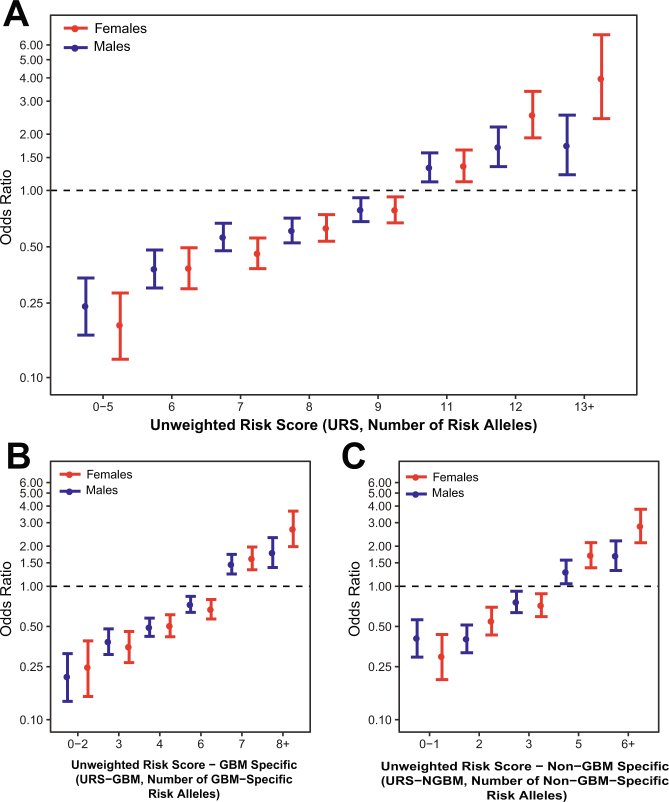
Odds ratios and 95% confidence intervals for unweighted risk (URS) score in (**A**) all glioma, (**B**) GBM-specific URS (URS-G) in GBM, and (**C**) and non-GBM-specific URS (URS-NGBM) for in non-GBM.

In order to estimate the cumulative effects of significant variants by sex, unweighted risk scores (URS) were calculated by summing all risk alleles for each individual using the 10 SNPs (rs12752552, rs9841110, rs10069690, rs11979158, rs55705857, rs634537, rs12803321, rs3751667, rs78378222, and rs2297440) found to be significantly associated with glioma in this analysis. GBM (URS-GBM) and non-GBM (URS-NGBM) specific URS were calculated only using sets of 6 SNPs in this set that were significantly associated with these histologies (URS-GBM: rs9841110, rs10069690, rs11979158, rs634537, rs78378222, and rs2297440, and URS-NGBM: rs10069690, rs55705857, rs634537, rs12803321, rs78378222, and rs2297440). See Methods for additional information on score calculation. Median URS, URS-GBM, and URS-NGBM were significantly different (p < 0.0001) between cases and controls in both males and females in all histology groups (Supplemental Fig. 5). There was no significant difference in median risk scores between male and female cases for any histology group. Glioma risk increased with increasing number of alleles in both males and females for the 10 SNPs included in the overall URS, as well as the 6 SNPs in the URS-GBM and 6 SNPs in URS-NGBM (Fig. 6, Supplemental Table 4). Risk was higher in females (OR = 3.97 [95% CI = 2.42–6.80]) as compared to males (OR = 1.74 [95% CI = 1.21–2.53]) in all glioma for individuals for with 13–16 alleles, though the difference between these estimates were not statistically significant. Risk was also higher among females (OR = 2.69 [95% CI = 1.98–3.66]) as compared to males (OR = 1.79 [95% CI = 1.38–2.32]) in GBM for individuals with 8–11 risk alleles, as well as in non-GBM for individuals with 6–11 risk alleles (females: OR = 2.83 [95% CI = 2.12–3.78], males: OR = 1.70 [95% CI = 1.31–2.19]), though the difference between these estimates were not statistically significant. The estimates may underestimate actual risk due to varying effect sizes and alleles frequencies between risk variants.

## Discussion

This is the first analysis of inherited risk variants in sporadic glioma focused specifically on sex differences, and the first agnostic unbiased scan for glioma risk variants on the X and Y sex chromosomes. One SNP at the 7p11.2 locus (rs11979158) showed significant association in males only, in both all glioma and GBM (Table 2, see Supplemental Table 5 and Supplemental Fig. 6 for study-specific estimates). This variant is within one of two previously identified independent glioma risk loci located near epidermal growth factor receptor (*EGFR*) and is most strongly associated with risk for GBM^4,17^. Though *EGFR* is implicated in many cancer types and is a target for many anti-cancer therapies, this risk locus has not been previously associated with any other cancer type. While estrogen has been demonstrated to interact with *EGFR* as well as other growth factors, previous epidemiological studies have not consistently found an association between proxy markers for endogenous estrogen exposure and decreased glioma risk^18^. Cell intrinsic, hormone independent sex differences in EGF effects have been observed in a murine model of gliomagenesis, where EGF treatment was transforming for male but not female astrocytes that had been rendered null for neurofibromin and p53 function^19^. While this SNP was not genotyped on the germline genotyping array used for TCGA, a SNP in strong LD with rs11979158 (rs7785013, D’ = 1, r^2^ = 1 in CEU^16^) was evaluated using a case-only approach. The association was not statistically significant in any histology group, but a similar trend to that observed in the overall meta-analysis in sex-specific RAF was observed in both the overall GBM and the *IDH1/2* wild type groups.

The association at 8q24.21 (rs55705857) is the strongest that has been identified by glioma GWAS to date^4^, with an odds ratio of 1.99 (95% CI = 1.85–2.13, p = 9.53 × 10^−79^) in glioma overall, and an odds ratio of 3.39 (95% CI = 3.09–3.71, p = 7.28 × 10^−149^) in non-GBM (see Supplemental Table 5 and Supplemental Fig. 7 for study-specific estimates). The identified SNP, rs55705857, is located in an intergenic region near coiled-coil domain containing 26 (*CCDC26*, a long non-coding RNA). This analysis found a stronger association in females than males in all glioma and non-GBM, where female odds ratio estimates are ~2 × those of males (Table 2). ORs were higher in women than men in all studies included in the analysis (see Supplemental Tables 5 and 6 for study-specific estimates and MAF). A sensitivity analysis was conducted to assess the effect of study heterogeneity on this estimate in non-GBM using only the GICC, MDA-GWAS, and GliomaScan datasets. The exclusion of SFAGS-GWAS did not substantially change the results (Main analysis p_D_ = 1.20 × 10^−6^ and sensitivity p_D_ = 1.49 × 10^−5^). A histology-specific analysis found a similar sex differences in ORs for rs55705957 for both non-GBM astrocytoma, and oligodendroglioma (Table 3, see Supplemental Table 7 for study-specific estimates). This variant is strongly associated with *IDH1*/*2* mutant and 1p/19q codeleted glioma tumors, but data on these molecular markers was not available for the four GWAS datasets used^20,21^. The TCGA GBM and LGG datasets^22–24^ were used to assess potential sex differences in frequency of *IDH1/2* mutation within histologies. Approximately the same proportion of males as females with histologically confirmed GBM had *IDH1/2* mutations (5.2% vs 6.0%, respectively, Supplemental Fig. 2). While this SNP was not genotyped on the germline genotyping array used for TCGA, a SNP in weak LD with rs55705857 (rs4636162, D’ = 1; r^2^ = 0.104, in CEU^16^) was evaluated using a case-only approach. There was no significant association in the overall meta-analysis for this SNP, and the association in the analysis of TCGA cases was not statistically significant in any group.

A large region in 3p21.31 was identified that was associated with all glioma and GBM in females only (Table 2, see Supplemental Table 5 and Supplemental Fig. 8 for study-specific estimates). The strongest association in this region was rs9841110, an intronic variant located upstream of dystroglycan 1 (*DAG1*) within an enhancer region. While this SNP was not genotyped on the germline genotyping array used for TCGA, a SNP in strong LD with rs9841110 (rs9814873, D’ = 1, r^2^ = 1 in CEU^16^) was able to be evaluated using a case-only approach. The association was not statistically significant in any group, but a similar trend in RAF was observed in the overall GBM and *IDH1/2* wild type groups. Though this region has not previously been associated with glioma, previous GWAS have detected associations at 3p21.31 for a large variety of traits, including several autoimmune diseases as well as increased age at menarche^25–28^. If increased lifetime estrogen exposure decreases glioma risk, as some have hypothesized, it is reasonable that variants which increase age at menarche (potentially decreasing total lifetime estrogen exposure) may increase glioma risk in females. Due to the complexity of measuring lifetime estrogen exposure (which is affected by age at menarche, age at menopause, parity, breast feeding patterns, and estrogen replacement therapy post-menopause) it is difficult to determine the ‘true’ effect that this exposure might have on glioma risk.

As compared to a model containing age at diagnosis and sex alone, the three SNPs (rs55705857, rs9841110 and rs11979158) identified as having sex-specific effects explain an additional 1.4% of trait variance within the GICC set. The variance explained by these SNPs varies by histology (0.6% in GBM, and 3.3% in Non-GBM). The variance explained by the addition of these three SNPs was higher in females for all glioma (1.3% in males and 2.2% in females), and non-GBM glioma (2.3% in males and 5.3% in females), and slightly higher in males for GBM (0.9% in males and 0.7% in females). Unweighted risk scores (URS) were generated to compare the cumulative effects of glioma risk variants by sex by summing all risk alleles using the 10 SNPs found to be significantly associated with glioma in this analysis. GBM (URS-GBM) and non-GBM (URS-NGBM) specific URS were calculated using sets of six SNPs in this set that were associated with significantly associated with each histology. Individuals with lower numbers of risk alleles had significantly lower odds of glioma, and those with higher numbers of alleles had increased odds of glioma, with statistically significant trends in each histology group. Males and females with low risk scores had similar odds of glioma, while females had increased odds in the upper strata of scores as compared to males. Development of risk scores that weight alleles by effect size, and use sex-specific estimates for variants for which effect size varies by sex (such as 7p11.2 and 8q24.21), may lead to better predictive values.

While often not included in GWAS, sex-stratified analyses can reveal genetic sources of sexual dimorphism in risk^9,10^. Sex-stratified analyses not only contribute to understanding of sources of sex difference in incidence, but may also suggest mechanisms and pathways disease development that vary by sex. Sex variation in genetic susceptibility to disease is likely not due to sex differences in actual DNA sequence, but is thought to be the result of sex-specific regulatory functions^29–31^. In addition to genetic sources of difference, there are likely several additional factors acting in combination which contribute to sex differences in glioma incidence. Sex differences in disease can also be linked to in-*utero* development, during which time gene expression and risk phenotypes are patterned through the action of X chromosome alleles that escape inactivation and genes on the non-pseudo-autosomal component of the Y chromosome, as well as the epigenetic effects of in utero testosterone^32^. A previous analysis estimating heritability of brain and CNS tumors by sex using twins attempted to estimate sex-specific relative risks, but these analyses were limited by a small sample size^33^. Further investigation of the inheritance patterns of familial glioma by sex may also provide additional information about sex differences in this disease.

There are several limitations to this analysis. Individuals included in these datasets were recruited during different time periods from numerous institutions, with no central review of pathology. Molecular tumor markers were unavailable for all datasets, and as a result classifications are based on the treating pathologist using the prevailing histologic criteria at time of diagnosis. The variant at 8q24.21 has been shown to have significant association with particular molecular subtypes, and without molecular data it was not possible to determine whether the observed result is an artifact of varying molecular features by sex. Oligodendroglioma as a histology is highly enriched for *IDH1/2* and 1p/19q co-deleted tumors (117/174, or ~67% within TCGA) and it is therefore likely that the analysis using only tumors classified as oligodendroglioma captured most of this molecular subtype. Males and females within histology groups have different frequencies of *IDH1/2* mutation^22^, which may have confounded the estimates for 8q24.21. The TCGA dataset was used to explore sex differences in allele frequency within molecular groups, but none of the identified SNPs were able to be directly validated within this set; however SNPs in strong LD were evaluated except for in 8q24.21. The 8q24.21 region is not well characterized on the array used for the TCGA genotyping, and as a result this region imputed poorly. No proxy SNP in strong LD with rs55705857 was able to be identified. Similar trends in RAF to those observed in the overall meta-analysis were seen in the TCGA set, though these differences were not statistically significant. Further interrogation in datasets with molecular classification where direct genotyping of these regions is warranted in order to confirm the sex-specific associations observed in this analysis.

## Conclusions

Sex and other demographic differences in cancer susceptibility can provide important clues to etiology, and these differences can be leveraged for discovery in genetic association studies. This analysis identified potential sex-specific effects in 2 previous identified glioma risk loci (7p11.2, and 8q24.21), and 1 newly identified autosomal locus (3p21.31). Odds ratios for the highest strata of an unweighted risk score calculated by summing total risk alleles was higher in females as compared to males in all three histology groups. These significant differences in effect size may be a result of differing biological function of these variants by sex due to biological sex differences, or interaction between these variants and unidentified risk factors that vary in prevalence or effect by sex.

## Materials and Methods

### Study cohorts

This study was approved locally by the institutional review board (IRB) at University Hospitals Cleveland Medical Center and by each participating study site’s IRB. Written informed consent was obtained from all participants. All research was performed in accordance with relevant guidelines and regulation. In this study, data was combined from four prior glioma GWAS: Glioma International Case-Control Study (GICC), San Francisco Adult Glioma Study GWAS (SFAGS-GWAS), MD Anderson Glioma GWAS (MDA-GWAS), and National Cancer Institute’s GliomaScan (Fig. 4A)^4,11–14^. The SFAGS-GWAS includes controls from the Illumina iControls dataset, and MDA-GWAS includes controls from Cancer Genetic Markers of Susceptibility (CGEMS) breast and prostate studies^34–36^. Details of data collection and classification are available in previous publications^4,11–14^.

### Genotyping and imputation of GWAS datasets

GICC cases and controls were genotyped on the Illumina Oncoarray^37^. The array included 37,000 beadchips customized to include previously-identified glioma-specific candidate single nucleotide polymorphisms (SNPs). SFAGS-GWAS cases and some controls were genotyped on Illumina’s HumanCNV370-Duo BeadChip, and the remaining controls were genotyped on the Illumina HumanHap300 and HumanHap550. MDA-GWAS cases were genotyped on the Illumina HumanHap610 and controls using the Illumina HumanHap550 (CGEMS breast^34,36^) or HumanHap300 (CGEMS prostate^35^). GliomaScan cases were genotyped on the Illumina 660 W, while controls were selected from cohort studies and were genotyped on Illumina 370D, 550 K, 610Q, or 660 W (See Rajaraman *et al*. for specific details of genotyping)^14^. Details of DNA collection and processing are available in previous publications^4,12–14^. Individuals with a call rate (CR) <99% were excluded, as well as all individuals who were of non-European ancestry (<80% estimated European ancestry using the FastPop^38^ procedure developed by the GAMEON consortium). For all apparent first-degree relative pairs were removed (identified using estimated identity by descent [IBD] ≥ .5), for example, the control was removed from a case-control pair; otherwise, the individual with the lower call rate was excluded. SNPs with a call rate < 95% were excluded as were those with a minor allele frequency (MAF) <0.01, or displaying significant deviation from Hardy-Weinberg equilibrium (HWE) (p < 1 × 10^−5^). Additional details of quality control procedures have been previously described in Melin *et al*.^4^. All datasets were imputed separately using SHAPEIT v2.837 and IMPUTE v2.3.2 using a merged reference panel consisting of data from phase three of the 1,000 genomes project and the UK10K^39–44^.

TCGA cases were genotyped on the Affymetrix Genomewide 6.0 array using DNA extracted from whole blood (see previous manuscript for details of DNA processing^23,24^), and underwent standard GWAS QC, and duplicate and related individuals within datasets have been excluded^4^. Ancestry outliers were identified in TCGA using principal components analysis in plink 1.9^45^. Resulting files were imputed using Eagle 2 and Minimac3 as implemented on the Michigan imputation server (https://imputationserver.sph.umich.edu) using the Haplotype Reference Consortium Version r1.1 2016 as a reference panel^46–48^. Somatic characterization of TCGA cases was obtained from the final dataset used for the TCGA pan-glioma analysis^22^, and classification schemes were adopted from Eckel-Passow, *et al*.^49^ and Ceccarelli, *et al*.^22^.

### Sex-stratified scan of the autosomal chromosomes

The data were analyzed using sex-stratified logistic regression models in SNPTEST for all SNPs on autosomal chromosomes within 500 kb of previously identified risk loci, and/or those found to be nominally significant (p < 5 × 10^−4^) in a previous meta-analysis (Fig. 2A)^4,50^. Sex-specific betas (β_M_ and β_F_), standard errors (SE_M_ and SE_F_), and p-values (p_M_ and p_F_) were generated using sex-stratified logistic regression models that were adjusted for number of principal components found to significant differed between cases and controls within each study in a previous meta-analysis^4,50^. Genomic inflation factors were calculated After excluding SNPs with MAF < 0.05, INFO score < 0.7, and that significantly violated Hardy-Weinberg equilibrium in controls (p < 5 × 10^−8^), genomic inflation factors (Males: GICC: λ_adjusted_ = 1.04, SFAGS-GWAS: λ_adjusted_ = 1.01 MDA-GWAS: λ_adjusted_ = 1.02; Gliomascan: λ_adjusted_ = 1.01. Females: GICC: λ_adjusted_ = 1.03; SFAGS-GWAS: λ_adjusted_ = 1.02; MDA-GWAS: λ_adjusted_ = 1.04; Gliomascan: λ_adjusted_ = 1.01).

### Estimation of sex difference and test of statistical significance

β_D_ and SE_D_ were estimated using the sex-specific betas and standard errors separately for each dataset, as follows:

1 $${\beta }_{D}={\beta }_{M}-{\beta }_{F}$$

2 $$S{E}_{D}=\sqrt{S{E}_{M}^{2}+S{E}_{F}^{2}}$$

The difference between the groups was then tested using a z test^51,52^. Sex-stratified results and differences estimates from the four studies were separately combined via inverse-variance weighted fixed effects meta-analysis in META^53^. See Fig. 2A for schematic of autosomal analysis methods. Case only-analyses were performed for SNPs found to be significant in agnostic analyses using sex as outcome for all glioma, GBM, and non-GBM by study and betas and standard errors were combined via inverse-variance weighted fixed effects meta-analysis in META^53^.

### Sex chromosome analysis

X and Y chromosome data were available from GICC set only. Males and females were imputed separately for the X chromosome using the previously described merged reference panel. X chromosomes were analyzed using logistic regression model in SNPTEST module ‘newml’ assuming complete inactivation of one allele in females, and males are treated as homozygous females (Fig. 2B**)**. For prioritized SNPs in the combined model, sex-specific effect estimates were generated using stratified logistic regression models. Y chromosome data were analyzed using logistic regression in SNPTEST (Fig. 2B)^54^. Figures were generated using LocusZoom and R 3.3.2 using GenABEL, qqman, and ggplot^55–59^.

### Analysis of TCGA germline and somatic data

Only newly diagnosed cases from TCGA GBM and LGG with no neo-adjuvant treatment or prior cancer were used. Demographic characteristics, molecular classification and somatic alterations data was obtained from Ceccarelli, *et al*.^22^. Chi-square tests were used to compare the frequency of somatic alterations between age groups. SNPs found to be nominally significant (p < 5 × 10^−4^) in a previous 8 study meta-analysis^4^, with imputation quality ≥ 0.7 were identified within the TCGA genotype data and D’ and r^2^ values in CEU were used to select proxy SNPs^16^. Using these SNPs, a case-only analysis using sex as a binary phenotype was conducted using logistic regression in SNPTEST assuming an additive model to estimate beta, standard error, and p values^50^. Results were considered significant at p < 0.003 (Bonferroni correction for 15 tests, for the three assessed loci in each of five histology groups).

### Calculation of unweighted genetic risk scores

In order to estimate the cumulative effects of significant variants by sex, histology-specific unweighted risk scores were calculated using the SNPs found to be significantly associated with each outcome. Data from all four studies was merged, and any imputed genotypes with genotype probability >0.8 were converted to hard calls. An overall unweighted risk score (URS) was generated using the sum of risk alleles at rs12752552, rs9841110, rs10069690, rs11979158, rs55705857, rs634537, rs12803321, rs3751667, rs78378222, and rs2297440. As risk alleles are known to have histology specific associations^4^, histologic specific scores were generated for GBM and non-GBM using only the SNPs found to have a significant association with each histology. GBM-specific URS (URS-G) was calculated by summing the number of risk alleles at rs9841110, rs10069690, rs11979158, rs634537, rs78378222, and rs2297440. Non-GBM-specific (URS-N) specific URS was calculated by summing the number of risk alleles at rs10069690, rs55705857, rs634537, rs12803321, rs78378222, and rs2297440. Unweighted risk scores (URS) were calculated by summing all risk alleles for each individual. Differences in median scores between groups using were tested using Wilcoxon rank sum tests. Scores were compared against the median score for each set (URS: ten alleles, URS-GBM: six alleles, URS-NGBM: four alleles). Odds ratios and 95% confidence intervals for each level of the score using sex-stratified logistic regression adjusted for age at diagnosis (for controls where only an age range was available, the mean value of the range was used), where each score was compared to the median score within the entire population as described in Shete *et al*.^13^.

### Calculation of trait variance explained by SNPs with sex-specific effects

In order to determine whether the identified SNPs with sex-specific effects more accurate estimate odds of glioma than sex alone, logistic regression models were used to estimate odds of all glioma, GBM, and non-GBM glioma based on sex using the GICC data only. Proportion of variance in odds of glioma explained by sex-specific SNPs was calculated using R^2^ estimated using the log likelihood of the null model (sex, age at diagnosis, and the first two principal components only) and the full model (including identified SNPs, rs9841110, rs11979158, rs55705857)^60^, calculated as follows:

3 $${R}^{2}=1-\frac{\mathrm{log}({L}_{full})}{\mathrm{log}({L}_{null})}$$

Proportion of variance explained was also calculated separately by sex for each histology (null model adjusted for age at diagnosis, and the first two principal components only).

## Electronic supplementary material

Supplemental Tables 1-7, Supplemental Figures 1-8
