# Supplementary material for: Sex-specific glioma genome-wide association study identifies new risk locus at 3p21.31 in females, and finds sex-differences in risk at 8q24.21
Source: Sci Rep. 2018 May 9;8:7352. doi: 10.1038/s41598-018-24580-z (PMC5943590; doi:10.1038/s41598-018-24580-z)

Sex-specific glioma genome-wide association study identifies new risk locus at 3p21.31 in females, and finds sex-differences in risk at 8q24.21

Quinn T. Ostrom[1-3], Ben Kinnersley[4], Margaret R. Wrensch[5], Jeanette E. Eckel-Passow[6], Georgina Armstrong[1], Terri Rice[5], Yanwen Chen[2], John K. Wiencke[5], Lucie S. McCoy[5], Helen M. Hansen[5], Christopher I. Amos[7], Jonine L. Bernstein[8], Elizabeth B. Claus[9,10], Dora Il'yasova[11-13], Christoffer Johansen[14], Daniel H. Lachance[15], Rose K. Lai[16,17], Ryan T. Merrell[18], Sara H. Olson[8], Siegal Sadetzki[19,20], Joellen M. Schildkraut[21], Sanjay Shete[22], Joshua B. Rubin[23], Justin D. Lathia[24], Michael E. Berens[25], Ulrika Andersson[26], Preetha Rajaraman[27], Stephen J. Chanock[27,28], Martha S. Linet[27], Zhaoming Wang[27,28], Meredith Yeager[27,28] (on behalf of the GliomaScan consortium^), Richard S. Houlston[4], Robert B. Jenkins[29], Beatrice Melin[26], Melissa L. Bondy[1], Jill. S. Barnholtz-Sloan[2]

1. Department of Medicine, Section of Epidemiology and Population Sciences, Dan L. Duncan Comprehensive Cancer Center, Baylor College of Medicine, Houston, Texas, United States of America.
2. Case Comprehensive Cancer Center, Case Western Reserve University School of Medicine, Cleveland, Ohio, United States of America.
3. Department of Population and Quantitative Heath Sciences, Case Western Reserve University School of Medicine, Cleveland, Ohio, United States of America.
4. Division of Genetics and Epidemiology, The Institute of Cancer Research, Sutton, Surrey, United Kingdom.
5. Department of Neurological Surgery and Institute of Human Genetics, School of Medicine, University of California, San Francisco, San Francisco, California, United States of America.
6. Division of Biomedical Statistics and Informatics, Mayo Clinic College of Medicine, Rochester, Minnesota, United States of America.
7. Institute for Clinical and Translational Research, Dan L. Duncan Comprehensive Cancer Center, Baylor College of Medicine, Houston, Texas, United States of America.
8. Department of Epidemiology and Biostatistics, Memorial Sloan Kettering Cancer Center, New York, New York, United States of America.
9. School of Public Health, Yale University, New Haven, Connecticut, United States of America.
10. Department of Neurosurgery, Brigham and Women's Hospital, Boston, Massachusetts, United States of America.
11. Department of Epidemiology and Biostatistics, School of Public Health, Georgia State University, Atlanta, Georgia, United States of America.
12. Cancer Control and Prevention Program, Department of Community and Family Medicine, Duke University Medical Center, Durham, North Carolina, United States of America.
13. Duke Cancer Institute, Duke University Medical Center, Durham, North Carolina, United States of America.
14. Oncology clinic, Finsen Center, Rigshospitalet and Survivorship Research Unit, The Danish Cancer Society Research Center, Copenhagen, Denmark
15. Department of Neurology, Mayo Clinic Comprehensive Cancer Center, Mayo Clinic, Rochester, Minnesota, United States of America.
16. Department of Neurology, Keck School of Medicine, University of Southern California, Los Angeles, California, United States of America.
17. Department of Preventive Medicine, Keck School of Medicine, University of Southern California, Los Angeles, California, United States of America.
18. Department of Neurology, NorthShore University HealthSystem, Evanston, Illinois, United States of America.
19. Cancer and Radiation Epidemiology Unit, Gertner Institute, Chaim Sheba Medical Center, Tel Hashomer, Israel.
20. Department of Epidemiology and Preventive Medicine, School of Public Health, Sackler Faculty of Medicine, Tel-Aviv University, Tel-Aviv, Israel.
21. Department of Public Health Sciences, University of Virginia School of Medicine, Charlottesville, Virginia, United States of America.
22. Department of Biostatistics, University of Texas MD Anderson Cancer Center, Houston, Texas, United States of America.
23. Department of Pediatrics, Washington University School of Medicine, St. Louis, Missouri; Department of Neuroscience, Washington University School of Medicine, St. Louis, Missouri, United States of America.
24. Department of Stem Cell Biology and Regenerative Medicine, Cleveland Clinic Foundation, Cleveland, Ohio, United States of America.
25. Cancer and Cell Biology Division, The Translational Genomics Research Institute, Phoenix, Arizona, United States of America.
26. Department of Radiation Sciences, Faculty of Medicine, Umeå University, Umeå, Sweden
27. Division of Cancer Epidemiology and Genetics, National Cancer Institute, Rockville, Maryland, United States of America.
28. Core Genotyping Facility, National Cancer Institute, SAIC-Frederick, Inc, Gaithersburg, Maryland, United States of America.
29. Department of Laboratory Medicine and Pathology, Mayo Clinic Comprehensive Cancer Center, Mayo Clinic, Rochester, Minnesota, United States of America.

Supplementary Materials

Supplemental Table 1. Case-only odds ratios (OR), 95% confidence intervals (95% CI), and p values from meta-analysis and individual studies for rs11979158, rs55705857 and rs9841110 overall and by histology groupings.

Supplemental Table 2. Characteristics of individuals in The Cancer Genome Atlas, by study and sex.

Supplemental Table 3. Linkage disequilibrium measures, sex-stratified odds ratios, and 95% confidence intervals (95% CI), and p values from meta-analysis for marker SNPs selected within the Cancer Genome Atlas genotyping data .

Supplemental Table 4. Odds ratios and 95% confidence intervals for unweighted scores in all glioma, GBM, and non-GBM overall and by sex.

Supplemental Table 5. Info score, sex-specific odds ratios (OR), 95% confidence intervals (95% CI), and p values from meta-analysis and individual studies for rs11979158, rs55705857 and rs9841110 overall and by histology groupings.

Supplemental Table 6. Risk allele frequencies (RAF), for meta-analysis and individual studies for rs11979158, rs55705857 and rs9841110 overall and by histology groupings.

Supplemental Table 7. Info score, sex-specific odds ratios (OR), 95% confidence intervals (95% CI), and p values from meta-analysis and individual studies for rs11979158, rs55705857 and rs9841110 by specific non-GBM histologies.

Supplemental Figure 1. P values of SNPs between 48.8mb and 50mb on chromosome 3 in males for A) all glioma, B) GBM, and C) non-GBM, and in females for D) all glioma, E) GBM, and F) non-GBM

Supplemental Figure 2. Proportion of samples with *IDH1/2* mutation in the TCGA GBM and LGG datasets by sex, overall and stratified by study

Supplemental Figure 3. Proportion of samples by glioma subtype (based on *IDH1/2* mutation, 1p19q, and *TERT* mutation) in the TCGA GBM and LGG datasets by sex, overall and stratified by study

Supplemental Figure 4. Proportion of samples by pan-glioma methylation subgroups [23] in the TCGA GBM and LGG datasets by sex, overall and stratified by study

Supplemental Figure 5. Density of histology-specific unweighted risk score by sex and case/control status for A) URS in all glioma, B) URS in GBM, C) URS in non-GBM, D) URS-GBM in GBM, only and E) URS-NGBM in non-GBM only

Supplemental Figure 6. Sex-specific odds ratios and 95% CI from meta-analysis and by study for rs11979158 (7p11.2) for all glioma, GBM, and non-GBM

Supplemental Figure 7. Sex-specific odds ratios and 95% CI from meta-analysis and by study for rs55705857 (8q24.21) for all glioma, GBM, and non-GBM

Supplemental Figure 8. Sex-specific odds ratios and 95% CI from meta-analysis and by study for rs9841110 (3p21.31) for all glioma, GBM, and non-GBM

Supplemental Table 1. Case-only odds ratios (OR), 95% confidence intervals (95% CI), and p values from meta-analysis and individual studies for rs11979158, rs55705857 and rs9841110 overall and by histology groupings.

RSID (Locus)	Study	All Glioma			GBM			Non-GBM		
		P	OR (95% CI)	P_{het}	P	OR (95% CI)	P_{het}	P	OR (95% CI)	P_{het}
rs9841110 (3p21.31)	Meta-analysis	0.0520	0.93 (0.87-1.00)	0.2801	0.0428	0.91 (0.82-1.00)	0.3218	0.5709	0.97 (0.86-1.08)	0.7404
	GICC	0.2862	0.95 (0.87-1.04)	--	0.1032	0.90 (0.79-1.02)	--	0.9061	0.99 (0.86-1.14)	--
	SFAGS-GWAS	0.8498	0.98 (0.76-1.25)	--	0.5560	0.92 (0.69-1.22)	--	0.5600	1.17 (0.69-1.97)	--
	MDA-GWAS	0.8190	1.02 (0.84-1.24)	--	0.4452	1.11 (0.85-1.43)	--	0.5710	0.92 (0.68-1.23)	--
	Gliomascan	0.0115	0.82 (0.71-0.96)	--	0.0453	0.81 (0.65-1.00)	--	0.3430	0.87 (0.66-1.16)	--
rs11979158 (7p11.2)	Meta-analysis	0.0071	1.13 (1.03-1.24)	0.8834	0.2392	1.08 (0.95-1.22)	0.8130	0.0115	1.20 (1.04-1.39)	0.2339
	GICC	0.0924	1.11 (0.98-1.25)	--	0.5852	1.05 (0.88-1.24)	--	0.0764	1.18 (0.98-1.41)	--
	SFAGS-GWAS	0.4149	1.15 (0.82-1.62)	--	0.9093	1.02 (0.69-1.52)	--	0.1465	1.67 (0.84-3.33)	--
	MDA-GWAS	0.0820	1.24 (0.97-1.57)	--	0.8085	1.04 (0.75-1.45)	--	0.0214	1.5 (1.06-2.13)	--
	Gliomascan	0.2396	1.12 (0.93-1.36)	--	0.1607	1.22 (0.93-1.60)	--	0.7295	0.94 (0.65-1.35)	--
rs55705857 (8q24.21)	Meta-analysis	0.0012	1.20 (1.07-1.33)	0.0523	0.6513	1.04 (0.88-1.23)	0.1497	0.0084	1.23 (1.05-1.43)	0.0968
	GICC	0.0293	1.17 (1.02-1.34)	--	0.3163	0.89 (0.71-1.12)	--	0.0236	1.24 (1.03-1.49)	--
	SFAGS-GWAS	0.0522	1.56 (1.00-2.44)	--	0.2701	1.35 (0.79-2.32)	--	0.0573	2.23 (0.98-5.11)	--
	MDA-GWAS	0.4549	0.89 (0.66-1.21)	--	0.9869	1.00 (0.63-1.58)	--	0.3932	0.83 (0.55-1.27)	--
	Gliomascan	0.0024	1.47 (1.15-1.88)	--	0.0691	1.40 (0.97-2.02)	--	0.0511	1.53 (1.00-2.34)	

Abbreviations: GICC: Glioma International Case-Control Study; SFAGS-GWAS: San Francisco Adult Glioma Study GWAS; MDA-GWAS: MD Anderson GWAS

Supplemental Table 2. Characteristics of individuals in The Cancer Genome Atlas, by study and sex

Characteristic	Overall	Males	Females
**N**	786	458	328
GBM (% of total)	356 (45.3%)	221 (48.3%)	135 (41.2%)
Non-GBM (% of total)	430 (54.7%)	237 (51.7%)	193 (58.8%)
Mean Age (SD)	50.74 (15.55)	51.21 (15.59)	50.11 (15.49)
GBM - Mean Age (SD)	59.83 (12.89)	60.16 (12.33)	59.29 (13.79)
Non-GBM - Mean Age (SD)	43.23 (13.40)	42.85 (13.54)	43.69 (13.24)
IDH wild type (% of total)	373 (50.3%)	229 (52.8%)	144 (46.8%)
GBM (% of total)	297 (94.3%)	187 (94.0%)	110 (94.8%)
Non-GBM (% of total)	76 (17.8%)	42 (17.9%)	34 (17.7%)
IDH mutant (% of total)	369 (49.7%)	205 (47.2%)	164 (53.2%)
GBM (% of total)	18 (5.7%)	12 (6.0%)	6 (5.2%)
Non-GBM (% of total)	351 (82.2%)	193 (82.1%)	158 (82.3%)

Supplemental Table 3. Linkage disequilibrium measures, sex-stratified odds ratios, and 95% confidence intervals (95% CI), and p values from meta-analysis for marker SNPs selected within the Cancer Genome Atlas genotyping data

RSID (Locus) [Risk Allele]	Marker SNP		Histology	Results from Sex-Stratified 4 study meta-analysis				
	RSID [LD (CEU[a])]	Risk Allele		P_M	OR_M (95% CI)	P_F	OR_F (95% CI)	p_D
rs9841110 (3p21.31) [**C**/G]	rs9814873 [D'=1; r^2=1 (C=A,G=G)]	**A**/G	All glioma	0.6552	1.01 (0.95-1.08)	1.45×10^{-7}	1.22 (1.13-1.31)	0.0002
			GBM	0.4139	1.03 (0.96-1.11)	1.98×10^{-7}	1.26 (1.16-1.38)	0.0005
			Non-GBM	0.4526	0.97 (0.89-1.06)	0.0279	1.12 (1.01-1.23)	0.0259
rs11979158 (7p11.2) [**A**/G]	rs7785013 [D'=1; r^2=1 (A=G,G=A)]	**G**/A	All glioma	1.87×10^{-12}	1.34 (1.23-1.45)	0.0168	1.12 (1.02-1.23)	0.0046
			GBM	4.62×10^{-13}	1.41 (1.29-1.55)	1.11×10^{-4}	1.25 (1.12-1.39)	0.1073
			Non-GBM	2.18×10^{-5}	1.27 (1.14-1.42)	0.9088	0.99 (0.88-1.12)	0.0031
rs55705857 (8q24.21) [A/**G**]	rs4636162 [D’=1; r^2=0.104 (A=G,G=A)]	G/**A**	All glioma	0.0158	1.08 (1.01-1.15)	0.0018	1.12 (1.04-1.20)	0.4271
			GBM	0.6525	1.02 (0.95-1.09)	0.9398	1.00 (0.92-1.09)	0.8347
			Non-GBM	1.37×10^{-5}	1.21 (1.11-1.32)	6.07×10^{-8}	1.31 (1.19-1.45)	0.2134

a. As estimated using LD link (https://analysistools.nci.nih.gov/LDlink/)

Supplemental Table 4. Odds ratios and 95% confidence intervals for unweighted scores in all glioma, GBM, and non-GBM overall and by sex.

Histology group (Score)	Number of risk alleles	Males				Females			
		Cases (%)	Controls (%)	OR (95% CI)	P	Cases (%)	Controls (%)	OR (95% CI)	P
All glioma (URS[a])	0-5	46 (1.2%)	161 (4.3%)	0.24 (0.17-0.34)	$1.96x10^{-15}$	30 (1.2%)	175 (4.7%)	0.19 (0.13-0.28)	$1.52x10^{-15}$
	6	140 (3.7%)	286 (7.6%)	0.38 (0.30-0.48)	$6.20x10^{-16}$	105 (4.1%)	307 (8.3%)	0.38 (0.30-0.49)	$1.10x10^{-13}$
	7	411 (10.9%)	575 (15.3%)	0.56 (0.48-0.67)	$3.33x10^{-11}$	249 (9.7%)	597 (16.1%)	0.46 (0.38-0.56)	$1.09x10^{-15}$
	8	639 (17.0%)	815 (21.6%)	0.61 (0.52-0.71)	$2.04x10^{-10}$	457 (17.8%)	818 (22.1%)	0.63 (0.54-0.74)	$3.45 x10^{-8}$
	9	830 (22.1%)	826 (21.9%)	0.79 (0.68-0.91)	$1.60x10^{-3}$	557 (21.7%)	795 (21.4%)	0.79 (0.67-0.92)	$3.33 x10^{-3}$
	10	768 (20.4%)	621 (16.5%)	Ref	--	556 (21.7%)	621 (16.8%)	Ref	--
	11	543 (14.4%)	615 (16.3%)	1.33 (1.11-1.59)	$1.82x10^{-3}$	351 (13.7%)	292 (7.9%)	1.35 (1.11-1.65)	$2.35 x10^{-3}$
	12	247 (6.6%)	121 (3.2%)	1.71 (1.34-2.18)	$1.62x10^{-5}$	189 (7.4%)	82 (2.2%)	2.53 (1.91-3.39)	$1.91 x10^{-10}$
	13-16	110 (2.9%)	47 (1.2%)	1.74 (1.21-2.53)	$3.06x10^{-3}$	70 (2.7%)	20 (0.5%)	3.97 (2.42-6.80)	$1.42 x10^{-7}$
	Total	3,761	3,767	1.26 (1.23-1.30) per allele	Trend: $8.74x10^{-63}$	2564	3707	1.34 (1.30-1.38) per allele	Trend: $3.17x10^{-74}$
GBM (URS-GBM[b])	0-2	28 (1.2%)	181 (4.4%)	0.21 (0.14-0.31)	$1.10x10^{-13}$	20 (1.4%)	181 (4.4%)	0.25 (0.15-0.39)	$1.02x10^{-8}$
	3	128 (5.3%)	439 (10.6%)	0.39 (0.31-0.48)	$2.84x10^{-17}$	77 (5.3%)	485 (11.7%)	0.35 (0.27-0.46)	$2.77x10^{-14}$
	4	347 (14.4%)	945 (22.7%)	0.49 (0.42-0.58)	$2.23x10^{-18}$	217 (15.0%)	954 (23.0%)	0.51 (0.42-0.61)	$2.21x10^{-12}$
	5	631 (26.2%)	1,156 (27.8%)	0.73 (0.64-0.84)	$1.11x10^{-5}$	356 (24.7%)	1,181 (28.5%)	0.67 (0.57-0.80)	$3.88x10^{-6}$
	6	684 (28.4%)	920 (22.1%)	Ref	--	393 (27.3%)	877 (21.2%)	Ref	--
	7	430 (17.9%)	396 (9.5%)	1.46 (1.24-1.73)	$9.58x10^{-6}$	272 (18.9%)	374 (9.0%)	1.62 (1.33-1.97)	$1.54x10^{-6}$
	8-11	159 (6.6%)	120 (2.9%)	1.79 (1.38-2.32)	$9.47x10^{-6}$	107 (7.4%)	88 (2.1%)	2.69 (1.98-3.66)	$2.32x10^{-10}$
	Total	2,407	4,157	1.40 (1.35-1.46) per allele	Trend: $2.05x10^{-66}$	1442	4140	1.47 (1.40-1.54) per allele	Trend: $3.76x10^{-60}$
non-GBM (URS-NGBM[c])	0-1	59 (4.2%)	341 (8.8%)	0.41 (0.29-0.56)	$5.49x10^{-8}$	34 (3.1%)	346 (9.1%)	0.30 (0.20-0.44)	$1.09x10^{-9}$
	2	130 (9.2%)	715 (18.5%)	0.40 (0.32-0.51)	$1.06x10^{-13}$	129 (11.6%)	756 (19.9%)	0.55 (0.43-0.70)	$9.97x10^{-7}$
	3	340 (24.1%)	1,089 (28.2%)	0.76 (0.64-0.92)	$4.06x10^{-3}$	263 (23.7%)	1,145 (30.2%)	0.72 (0.59-0.88)	$1.25x10^{-3}$
	4	419 (29.7%)	979 (25.4%)	Ref	--	298 (26.8%)	921 (24.3%)	Ref	--
	5	288 (20.4%)	523 (13.5%)	1.28 (1.05-1.57)	$1.65x10^{-2}$	251 (22.6%)	477 (12.6%)	1.71 (1.38-2.12)	$1.08x10^{-6}$
	6-11	175 (12.4%)	213 (5.5%)	1.70 (1.31-2.19)	$5.19x10^{-5}$	137 (12.3%)	146 (3.9%)	2.83 (2.12-3.78)	$1.41x10^{-12}$
	Total	1,411	3,860	1.36 (1.29-1.42) per allele	Trend: $1.16x10^{-32}$	1,112	3,791	1.49 (1.41-1.58) per allele	Trend: $2.59x10^{-45}$

a. model includes age, and sum of risk alleles for rs12752552, rs9841110, rs10069690, rs11979158, rs55705857, rs634537, rs12803321, rs3751667, rs78378222, and rs2297440. Reference is 10 alleles.
b. model includes age and sum of risk alleles for rs9841110, rs10069690, rs11979158, rs634537, rs78378222, and rs2297440. Reference is 6 alleles.
c. model includes age and sum of risk alleles for rs10069690, rs55705857, rs634537, rs12803321, rs78378222, and rs2297440. Reference is 4alleles.

Supplemental Table 5. Info score, sex-specific odds ratios (OR), 95% confidence intervals (95% CI), and p values from meta-analysis and individual studies for rs11979158, rs55705857 and rs9841110 overall and by histology groupings.

RSID	Risk Allele	Histology	Study	Males								Sex Difference	
				$Info_M$	P_M	OR_M (95% CI)	P_{het}	$Info_F$	P_F	OR_F (95% CI)	P_{het}	P_D	P_{het}
rs9841110 (3p21.31)	**C**/G	**All glioma**	**Meta-analysis**		0.5885	1.02 (0.96-1.08)	0.0728		5.55×10^{-8}	1.22 (1.14-1.32)	0.2751	1.77×10^{-4}	0.6269
			GICC	1.00	0.3089	0.95 (0.87-1.05)		1.00	0.0123	1.15 (1.03-1.28)		0.0052	
			SFAGS-GWAS	1.00	0.016	1.25 (1.04-1.49)		1.00	0.0059	1.35 (1.09-1.67)		0.2857	
			MDA-GWAS	1.00	0.5538	1.05 (0.90-1.22)		1.00	0.0621	1.18 (0.99-1.40)		0.1586	
			Gliomascan	1.00	0.788	1.02 (0.90-1.16)		1.00	6.14×10^{-5}	1.34 (1.16-1.55)		0.0024	
		**GBM**	**Meta-analysis**		0.3429	1.04 (0.96-1.11)	0.1308		1.44×10^{-7}	1.27 (1.16-1.38)	0.2649	6.04×10^{-4}	0.633
			GICC	1.00	0.5104	0.97 (0.87-1.07)		1.00	0.0027	1.22 (1.07-1.39)		0.003	
			SFAGS-GWAS	1.00	0.021	1.26 (1.04-1.53)		1.00	0.0025	1.45 (1.14-1.85)		0.184	
			MDA-GWAS	1.00	0.5797	1.05 (0.88-1.27)		1.00	0.3271	1.11 (0.90-1.37)		0.3565	
			Gliomascan	1.00	0.4944	1.06 (0.90-1.23)		1.00	3.89×10^{-4}	1.38 (1.16-1.65)		0.0128	
		**Non-GBM**	**Meta-analysis**		0.4816	0.97 (0.89-1.06)	0.3776		0.016	1.13 (1.02-1.24)	0.5611	0.0186	0.7033
			GICC	1.00	0.1834	0.92 (0.82-1.04)		1.00	0.342	1.07 (0.94-1.21)		0.0547	
			SFAGS-GWAS	1.00	0.2235	1.21 (0.89-1.65)		1.00	0.619	1.10 (0.75-1.64)		0.3596	
			MDA-GWAS	1.00	0.7644	1.03 (0.85-1.26)		1.00	0.0567	1.26 (0.99-1.60)		0.1014	
			Gliomascan	1.00	0.6195	0.95 (0.77-1.17)		1.00	0.0795	1.22 (0.98-1.52)		0.0523	
rs11979158 (7p11.2)	**A**/G	**All glioma**	**Meta-analysis**		4.87×10^{-12}	1.33 (1.23-1.44)	0.1316		0.0187	1.12 (1.02-1.22)	0.772	0.0055	0.2849
			GICC	1.00	2.94×10^{-10}	1.47 (1.30-1.66)		1.00	0.1528	1.11 (0.96-1.27)		0.0013	
			SFAGS-GWAS	1.00	0.0256	1.32 (1.03-1.68)		1.00	0.2579	1.17 (0.89-1.54)		0.2631	
			MDA-GWAS	1.00	0.019	1.25 (1.04-1.51)		1.00	0.8186	1.02 (0.83-1.26)		0.0812	
			Gliomascan	1.00	0.0757	1.16 (0.98-1.37)		1.00	0.0705	1.18 (0.99-1.41)		0.4543	
		**GBM**	**Meta-analysis**		1.26×10^{-12}	1.40 (1.28-1.54)	0.4644		1.33×10^{-4}	1.24 (1.11-1.39)	0.9652	0.1184	0.6473
			GICC	1.00	1.14×10^{-9}	1.52 (1.33-1.73)		1.00	0.0254	1.22 (1.02-1.44)		0.0229	
			SFAGS-GWAS	1.00	0.0349	1.33 (1.02-1.73)		1.00	0.0784	1.32 (0.97-1.79)		0.484	
			MDA-GWAS	1.00	0.0371	1.27 (1.01-1.59)		1.00	0.1382	1.22 (0.94-1.59)		0.4067	
			Gliomascan	1.00	0.0078	1.32 (1.08-1.61)		1.00	0.0322	1.28 (1.02-1.59)		0.4175	
		**Non-GBM**	**Meta-analysis**		2.74×10^{-5}	1.27 (1.13-1.41)	0.3829		0.9014	0.99 (0.88-1.12)	0.1587	0.0034	0.1072
			GICC	1.00	3.48×10^{-5}	1.37 (1.18-1.60)		1.00	0.8557	0.98 (0.83-1.16)		0.0018	
			SFAGS-GWAS	1.00	0.3373	1.22 (0.82-1.82)		1.00	0.5226	0.85 (0.52-1.40)		0.1358	
			MDA-GWAS	1.00	0.1229	1.21 (0.95-1.54)		1.00	0.1696	0.82 (0.61-1.09)		0.0205	
			Gliomascan	1.00	0.6499	1.06 (0.82-1.38)		1.00	0.0901	1.27 (0.96-1.68)		0.1776	
rs55705857 (8q24.21)	A/**G**	**All glioma**	**Meta-analysis**		1.09×10^{-14}	1.56 (1.40-1.75)	0.0426		1.22×10^{-39}	2.45 (2.14-2.80)	0.6289	0	0.0662
			GICC	1.00	1.19×10^{-10}	1.68 (1.43-1.97)		1.00	1.63×10^{-19}	2.30 (1.92-2.76)		0.005	
			SFAGS-GWAS	1.00	0.4872	1.14 (0.78-1.67)		1.00	4.16×10^{-6}	3.18 (1.94-5.21)		6.44×10^{-4}	
			MDA-GWAS	1.00	2.67×10^{-6}	1.90 (1.46-2.49)		1.00	9.89×10^{-7}	2.46 (1.72-3.53)		0.1315	
			Gliomascan	1.00	0.0481	1.28 (1.00-1.62)		1.00	3.25×10^{-12}	2.60 (1.99-3.40)		5.59×10^{-5}	
		**GBM**	**Meta-analysis**		0.0344	1.17 (1.01-1.34)	0.8709		4.16×10^{-7}	1.61 (1.34-1.94)	0.0728	0.0066	0.0823
			GICC	1.00	0.051	1.22 (1.00-1.49)		1.00	0.0612	1.29 (0.99-1.69)		0.3702	
			SFAGS-GWAS	1.00	0.6649	1.10 (0.73-1.65)		1.00	0.0014	2.56 (1.44-4.57)		0.0094	
			MDA-GWAS	1.00	0.3057	1.21 (0.84-1.72)		1.00	0.063	1.56 (0.98-2.48)		0.197	
			Gliomascan	1.00	0.7125	1.06 (0.78-1.43)		1.00	8.59×10^{-5}	2.07 (1.44-2.97)		0.0027	
		**Non-GBM**	**Meta-analysis**		8.13×10^{-36}	2.66 (2.28-3.10)	0.026		1.85×10^{-65}	4.71 (3.94-5.63)	0.0514	3.46×10^{-7}	0.0662
			GICC	1.00	6.85×10^{-25}	2.81 (2.31-3.42)		1.00	1.42×10^{-39}	4.09 (3.32-5.05)		0.0051	
			SFAGS-GWAS	1.00	0.455	1.31 (0.65-2.66)		1.00	1.44×10^{-6}	12.68 (4.51-35.64)		1.89×10^{-4}	
			MDA-GWAS	1.00	3.17×10^{-12}	3.50 (2.46-4.97)		1.00	2.55×10^{-11}	6.38 (3.70-11.00)		0.0346	

			Gliomascan	1.00	0.0015	1.90 (1.28-2.82)		1.00	$1.82x10^{-14}$	6.17 (3.87-9.83)		$7.71x10^{-5}$	

Abbreviations: GICC: Glioma International Case-Control Study; SFAGS-GWAS: San Francisco Adult Glioma Study GWAS; MDA-GWAS: MD Anderson GWAS

Supplemental Table 6. Risk allele frequencies (RAF), for meta-analysis and individual studies for rs11979158, rs55705857 and rs9841110 overall and by histology groupings.

RSID	Risk Allele	Histology	Study	Males				Females			
				Cases		Controls		Cases		Controls	
				N	RAF	N	RAF	N	RAF	N	RAF
rs9841110 (3p21.31)	**C**/G	All glioma	Meta-analysis	4,831	0.307	5,176	0.313	3,206	0.291	5,417	0.337
			GICC	2,733	0.311	1,868	0.312	1,831	0.299	1,397	0.337
			SFAGS-GWAS	440	0.282	749	0.333	237	0.270	1,618	0.343
			MDA-GWAS	714	0.293	1,094	0.302	429	0.296	1,142	0.335
			Gliomascan	944	0.318	1,465	0.311	709	0.275	1,260	0.330
		GBM	Meta-analysis	2,835	0.304	5,176	0.313	1,682	0.283	5,417	0.337
			GICC	1,575	0.310	1,868	0.312	885	0.287	1,397	0.337
			SFAGS-GWAS	333	0.279	749	0.333	178	0.253	1,618	0.343
			MDA-GWAS	397	0.290	1,094	0.302	246	0.307	1,142	0.335
			Gliomascan	530	0.313	1,465	0.311	373	0.269	1,260	0.330
		Non-GBM	Meta-analysis	1,716	0.312	5,176	0.313	1,320	0.306	5,417	0.337
			GICC	1,036	0.317	1,868	0.312	862	0.314	1,397	0.337
			SFAGS-GWAS	107	0.290	749	0.333	59	0.322	1,618	0.343
			MDA-GWAS	317	0.297	1,094	0.302	183	0.281	1,142	0.335
			Gliomascan	256	0.325	1,465	0.311	216	0.294	1,260	0.330
		Astrocytoma (Non-GBM) (WHO grade II-III)	Meta-analysis	787	0.296	4,427	0.309	585	0.300	3,799	0.334
			GICC	494	0.299	1,868	0.312	400	0.307	1,397	0.337
			MDA-GWAS	155	0.274	1,094	0.302	88	0.288	1,142	0.335
			Gliomascan	138	0.312	1,465	0.311	97	0.279	1,260	0.330
		Oligodendroglioma (WHO grade II-III)	Meta-analysis	483	0.314	4,427	0.309	399	0.303	3,799	0.334
			GICC	310	0.321	1,868	0.312	266	0.312	1,397	0.337
			MDA-GWAS	95	0.289	1,094	0.302	54	0.241	1,142	0.335
			Gliomascan	78	0.314	1,465	0.311	79	0.316	1,260	0.330
rs11979158 (7p11.2)	**A**/G	All glioma	Meta-analysis	4,831	0.136	5,176	0.170	3,206	0.153	5,417	0.163
			GICC	2,733	0.135	1,868	0.186	1,831	0.149	1,397	0.164
			SFAGS-GWAS	440	0.125	749	0.154	237	0.141	1,618	0.151
			MDA-GWAS	714	0.137	1,094	0.166	429	0.164	1,142	0.166
			Gliomascan	944	0.146	1,465	0.163	709	0.160	1,260	0.173
		GBM	Meta-analysis	2,835	0.128	5,176	0.170	1,682	0.139	5,417	0.163
			GICC	1,575	0.129	1,868	0.186	885	0.135	1,397	0.164
			SFAGS-GWAS	333	0.122	749	0.154	178	0.126	1,618	0.151
			MDA-GWAS	397	0.134	1,094	0.166	246	0.140	1,142	0.166
			Gliomascan	530	0.128	1,465	0.163	373	0.151	1,260	0.173
		Non-GBM	Meta-analysis	1,716	0.145	5,176	0.170	1,320	0.167	5,417	0.163
			GICC	1,036	0.142	1,868	0.186	862	0.164	1,397	0.164
			SFAGS-GWAS	107	0.136	749	0.154	59	0.186	1,618	0.151
			MDA-GWAS	317	0.140	1,094	0.166	183	0.197	1,142	0.166
			Gliomascan	256	0.162	1,465	0.163	216	0.150	1,260	0.173
		Astrocytoma (Non-GBM) (WHO grade II-III)	Meta-analysis	787	0.134	4,427	0.066	585	0.136	3,799	0.057
			GICC	494	0.142	1,868	0.059	400	0.133	1,397	0.051
			MDA-GWAS	155	0.131	1,094	0.070	88	0.141	1,142	0.059
			Gliomascan	138	0.107	1,465	0.073	97	0.148	1,260	0.061
		Oligodendroglioma (WHO grade II-III)	Meta-analysis	483	0.168	4,427	0.066	399	0.207	3,799	0.057
			GICC	310	0.168	1,868	0.059	266	0.224	1,397	0.051
			MDA-GWAS	95	0.196	1,094	0.070	54	0.146	1,142	0.059
			Gliomascan	78	0.132	1,465	0.073	79	0.194	1,260	0.061
rs55705857 (8q24.21)	A/**G**	All glioma	Meta-analysis	4,831	0.096	5,176	0.066	3,206	0.112	5,417	0.055
			GICC	2,733	0.097	1,868	0.059	1,831	0.113	1,397	0.051
			SFAGS-GWAS	440	0.070	749	0.063	237	0.096	1,618	0.050
			MDA-GWAS	714	0.113	1,094	0.070	429	0.106	1,142	0.059
			Gliomascan	944	0.092	1,465	0.073	709	0.120	1,260	0.061
		GBM	Meta-analysis	2,835	0.072	5,176	0.066	1,682	0.075	5,417	0.055
			GICC	1,575	0.069	1,868	0.059	885	0.062	1,397	0.051
			SFAGS-GWAS	333	0.068	749	0.063	178	0.085	1,618	0.050
			MDA-GWAS	397	0.080	1,094	0.070	246	0.081	1,142	0.059
			Gliomascan	530	0.078	1,465	0.073	373	0.100	1,260	0.061
		Non-GBM	Meta-analysis	1,716	0.135	5,176	0.066	1,320	0.159	5,417	0.055
			GICC	1,036	0.140	1,868	0.059	862	0.168	1,397	0.051
			SFAGS-GWAS	107	0.073	749	0.063	59	0.130	1,618	0.050
			MDA-GWAS	317	0.154	1,094	0.070	183	0.139	1,142	0.059
			Gliomascan	256	0.116	1,465	0.073	216	0.152	1,260	0.061

		Astrocytoma (Non-GBM) (WHO grade II-III)	Meta-analysis	787	0.134	4,427	0.066	585	0.136	3,799	0.057
			GICC	494	0.142	1,868	0.059	400	0.133	1,397	0.051
			MDA-GWAS	155	0.131	1,094	0.070	88	0.141	1,142	0.059
		Oligodendroglioma (WHO grade II-III)	Gliomascan	138	0.107	1,465	0.073	97	0.148	1,260	0.061
			Meta-analysis	483	0.168	4,427	0.066	399	0.207	3,799	0.057
			GICC	310	0.168	1,868	0.059	266	0.224	1,397	0.051
			MDA-GWAS	95	0.196	1,094	0.070	54	0.146	1,142	0.059
			Gliomascan	78	0.132	1,465	0.073	79	0.194	1,260	0.061

Supplemental Table 7. Info score, sex-specific odds ratios (OR), 95% confidence intervals (95% CI), and p values from meta-analysis and individual studies for rs11979158, rs55705857 and rs9841110 by specific non-GBM histologies.

RSID	Histology	Study	Males				Females				Sex Difference	
			Info$_M$	P_M	OR$_M$ (95% CI)	P_{het}	Info$_F$	P_F	OR$_F$ (95% CI)	P_{het}	P_D	P_{het}
rs9841110 (3p21.31)	Astrocytoma (Non-GBM) (WHO grade II-III)	**Meta-analysis**	--	0.5304	1.04 (0.92-1.17)	0.7510	--	0.0407	1.15 (1.01-1.32)	0.549	0.2409	0.7420
		GICC	1.00	0.8819	1.01 (0.87-1.18)	--	1.00	0.3113	1.09 (0.92-1.29)	--	0.2594	
		MDA-GWAS	1.00	0.3388	1.14 (0.87-1.48)	--	1.00	0.2454	1.21 (0.88-1.68)	--	0.3799	
		Gliomascan	1.00	0.8658	1.02 (0.78-1.34)	--	1.00	0.0826	1.32 (0.96-1.80)	--	0.1134	
	Oligodendroglioma (WHO grade II-III)	**Meta-analysis**	--	0.4190	0.94 (0.81-1.09)	0.6940	--	0.0973	1.14 (0.98-1.34)	0.36	0.0649	0.7530
		GICC	1.00	0.2508	0.90 (0.74-1.08)	--	1.00	0.4020	1.09 (0.89-1.33)		0.0812	
		MDA-GWAS	1.00	0.8013	1.04 (0.75-1.45)	--	1.00	0.0500	1.51 (1.00-2.27)		0.0851	
		Gliomascan	1.00	0.9752	0.99 (0.70-1.41)	--	1.00	0.6171	1.09 (0.77-1.55)		0.3544	
rs11979158 (7p11.2)	Astrocytoma (Non-GBM) (WHO grade II-III)	**Meta-analysis**	--	0.0023	0.79 (0.68-0.92)	0.0560	--	0.9363	0.99 (0.83-1.18)	0.418	0.0500	0.0620
		GICC	1.00	$3.90x10^{-4}$	0.70 (0.58-0.85)	--	1.00	0.9938	1.00 (0.80-1.25)		0.0093	
		MDA-GWAS	1.00	0.1640	0.80 (0.58-1.10)	--	1.00	0.3965	1.19 (0.80-1.78)		0.0630	
		Gliomascan	1.00	0.4710	1.13 (0.81-1.59)	--	1.00	0.3097	0.82 (0.55-1.21)		0.1075	
	Oligodendroglioma (WHO grade II-III)	**Meta-analysis**		0.0221	0.81 (0.68-0.97)	0.8650	--	0.6561	1.05 (0.86-1.28)	0.262	0.0471	0.6470
		GICC	1.00	0.0475	0.79 (0.63-1.00)	--	1.00	0.6237	1.07 (0.83-1.37)		0.0450	
		MDA-GWAS	1.00	0.5593	0.89 (0.60-1.32)	--	1.00	0.1941	1.40 (0.84-2.33)		0.0830	
		Gliomascan	1.00	0.2618	0.78 (0.50-1.21)	--	1.00	0.3297	0.81 (0.53-1.24)		0.4496	
rs55705857 (8q24.21)	Astrocytoma (Non-GBM) (WHO grade II-III)	**Meta-analysis**	--	$1.19x10^{-21}$	2.87 (2.31-3.56)	0.0730	--	$2.15x10^{-28}$	4.64 (3.53-6.09)	0.237	0.0065	0.0370
		GICC	1.00	$1.72x10^{-18}$	3.34 (2.55-4.36)	--	1.00	$2.75x10^{-17}$	4.01 (2.91-5.53)		0.1947	
		MDA-GWAS	0.78	$6.98x10^{-5}$	2.77 (1.68-4.58)	--	0.75	$3.76x10^{-7}$	7.38 (3.41-15.96)		0.0185	
		Gliomascan	0.80	0.0509	1.68 (1.00-2.83)	--	0.80	$1.42x10^{-7}$	6.19 (3.14-12.22)		0.0014	
	Oligodendroglioma (WHO grade II-III)	**Meta-analysis**	--	$5.37x10^{-34}$	5.47 (4.16-7.19)	0.1030	--	$3.68x10^{-58}$	12.15 (8.96-16.48)	0.027	$6.60x10^{-5}$	0.0070
		GICC	1.00	$2.94x10^{-23}$	5.49 (3.92-7.68)	--	1.00	$3.10x10^{-40}$	10.24 (7.26-14.43)		0.0054	
		MDA-GWAS	0.79	$1.88x10^{-11}$	8.41 (4.52-15.66)	--	0.75	$3.97x10^{-6}$	11.57 (4.09-32.74)		0.3031	
		Gliomascan	0.79	0.0035	2.96 (1.43-6.15)	--	0.79	$1.53x10^{-16}$	36.02 (15.38-84.37)		$6.35x10^{-6}$	

Abbreviations: GICC: Glioma International Case-Control Study; SFAGS-GWAS: San Francisco Adult Glioma Study GWAS; MDA-GWAS: MD Anderson GWAS

Supplemental Figure 1. P values of SNPs between 48.8mb and 50mb on chromosome 3 in males for A) all glioma, B) GBM, and C) non-GBM, and in females for D) all glioma, E) GBM, and F) non-GBM

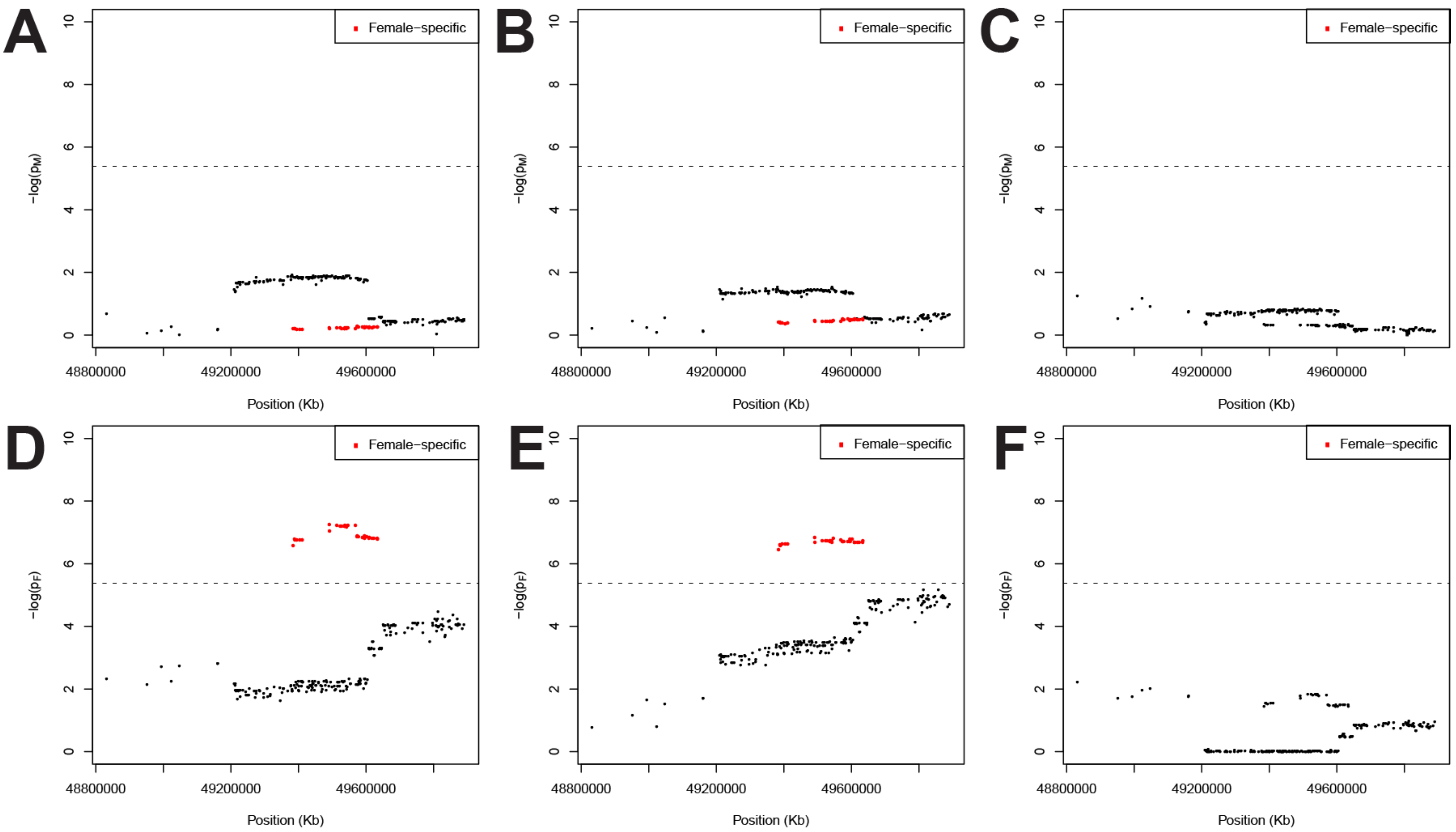

Supplemental Figure 2. Proportion of samples with *IDH1/2* mutation in the TCGA GBM and LGG datasets by sex, A) overall and B) stratified by study

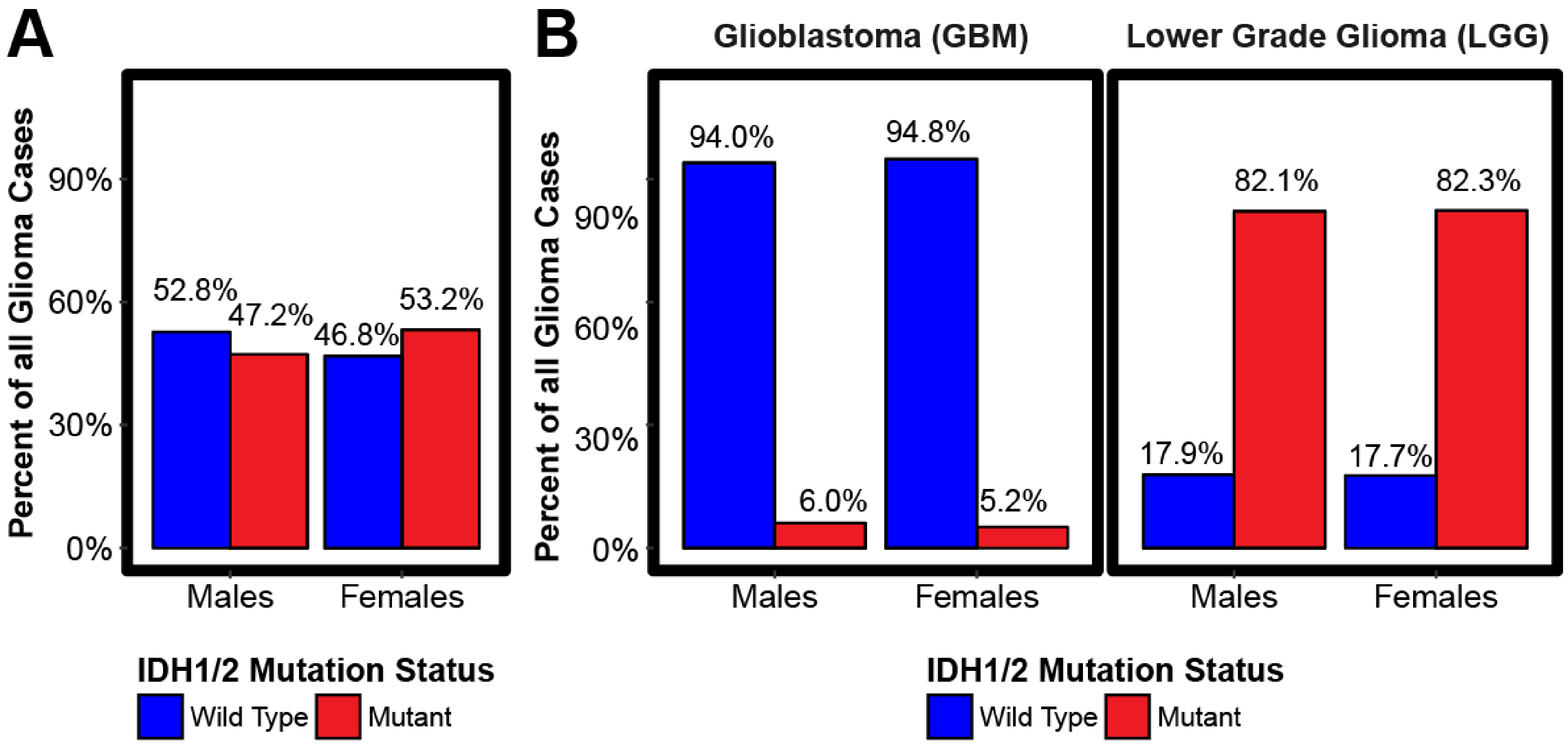

Supplemental Figure 3. Proportion of samples by glioma subtype (based on *IDH1/2* mutation, 1p19q, and TERT mutation) in the TCGA GBM and LGG datasets by sex, A) overall and B) stratified by study

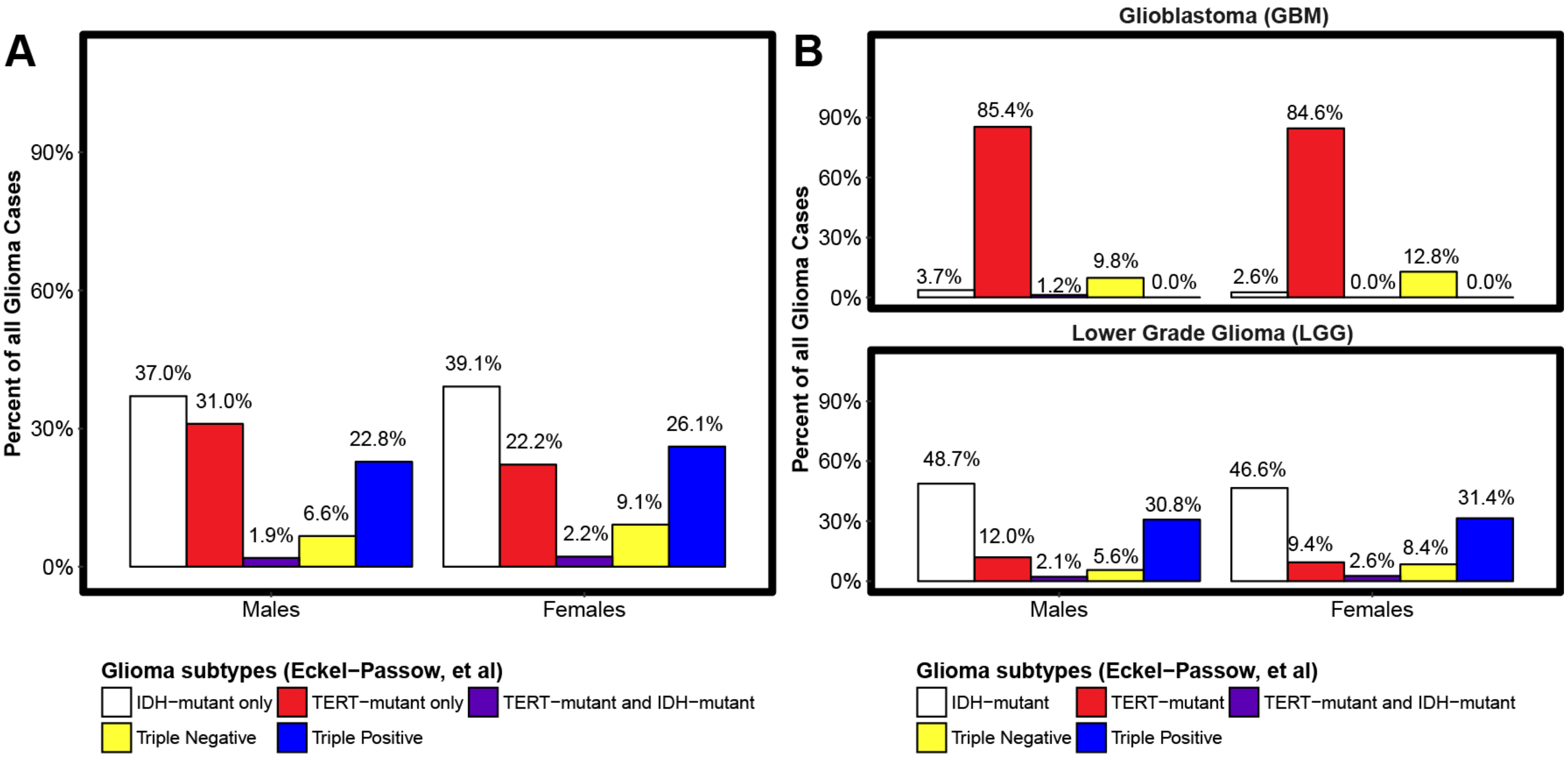

Supplemental Figure 4. Proportion of samples by pan-glioma methylation subgroups in the TCGA GBM and LGG datasets by sex, A) overall and B) stratified by study

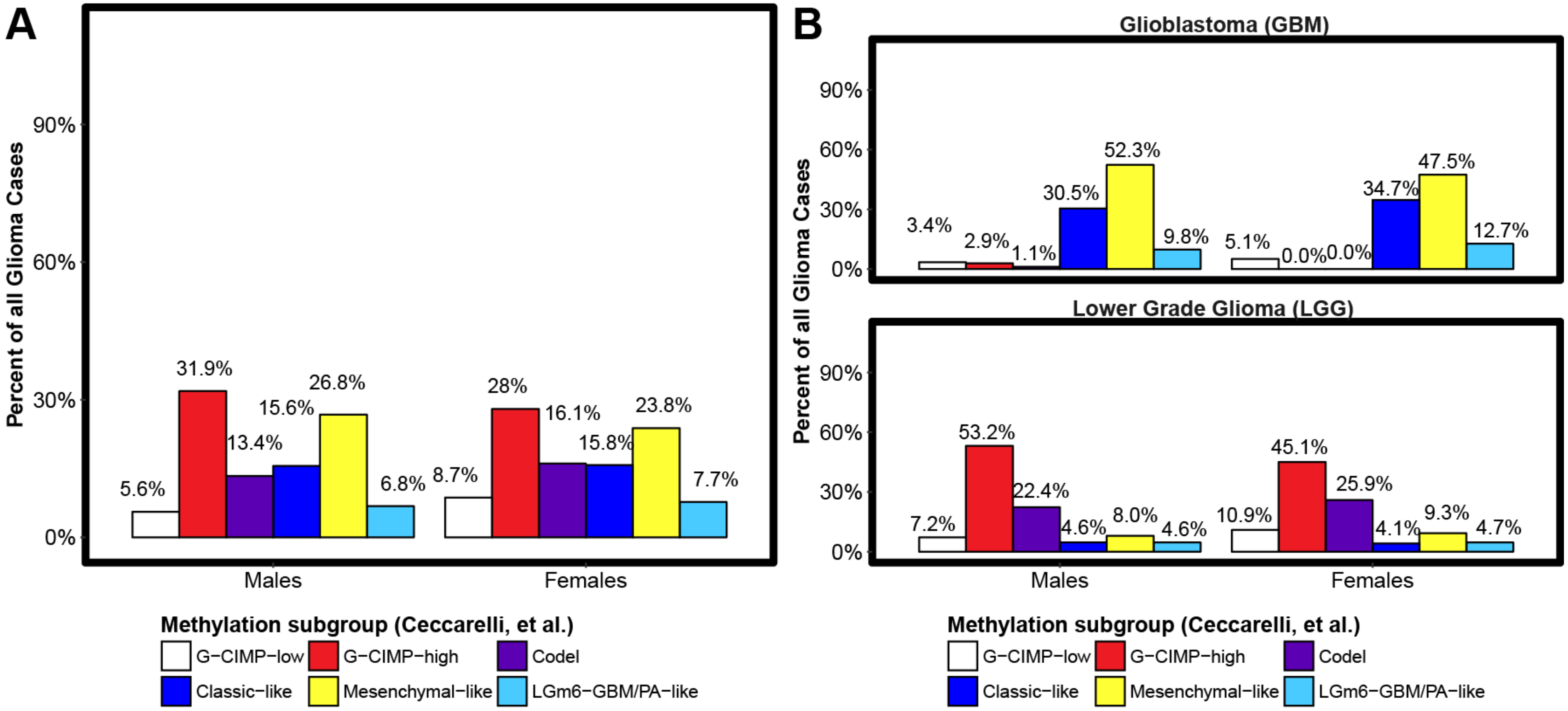

Supplemental Figure 5. Density of histology-specific unweighted risk score by sex and case/control status for A) URS in all glioma, B) URS in GBM, C) URS in non-GBM, D) URS-GBM in GBM, only and E) URS-NGBM in non-GBM only

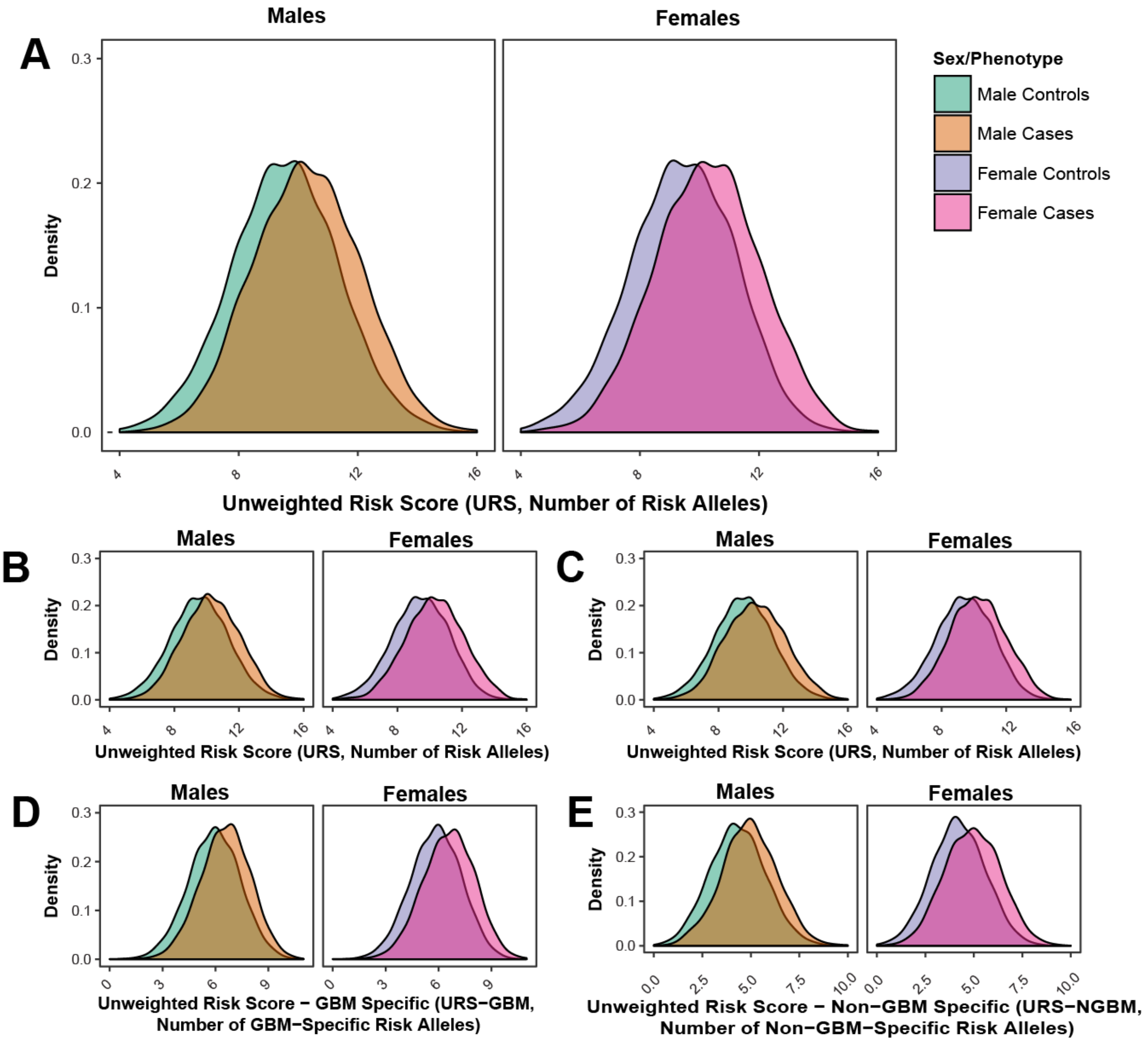

Supplemental Figure 6. Sex-specific odds ratios and 95% CI from meta-analysis and by study for rs11979158 (7p11.2) for all glioma, GBM, and non-GBM

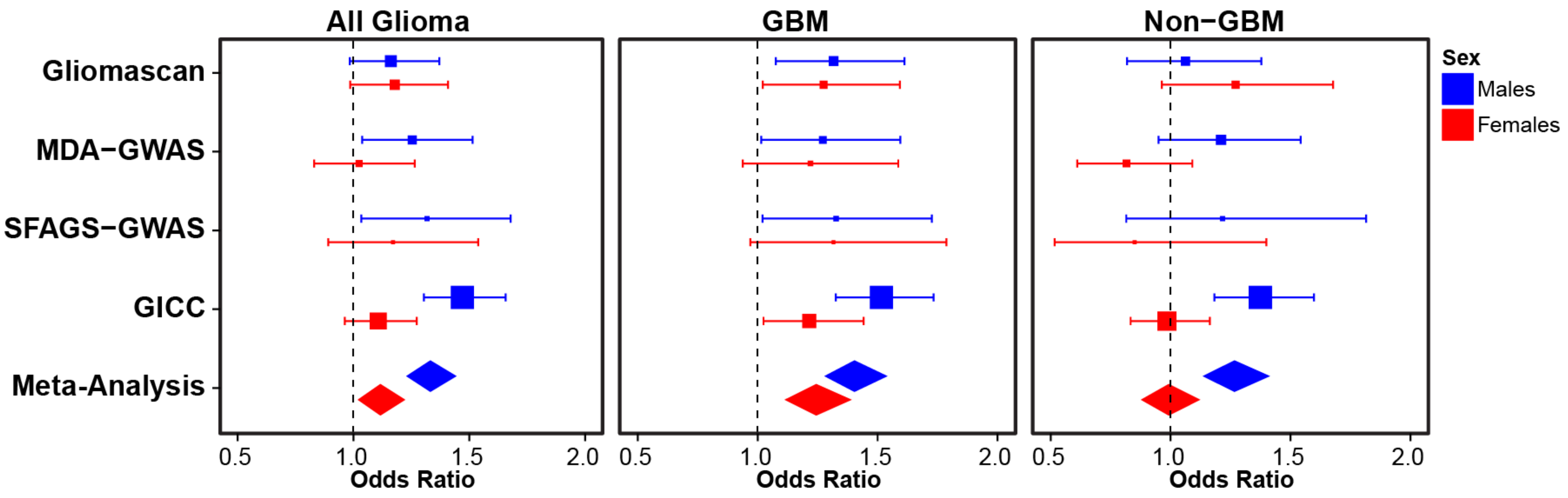

Supplemental Figure 7. Sex-specific odds ratios and 95% CI from meta-analysis and by study for rs55705857 (8q24.21) for all glioma, GBM, and non-GBM

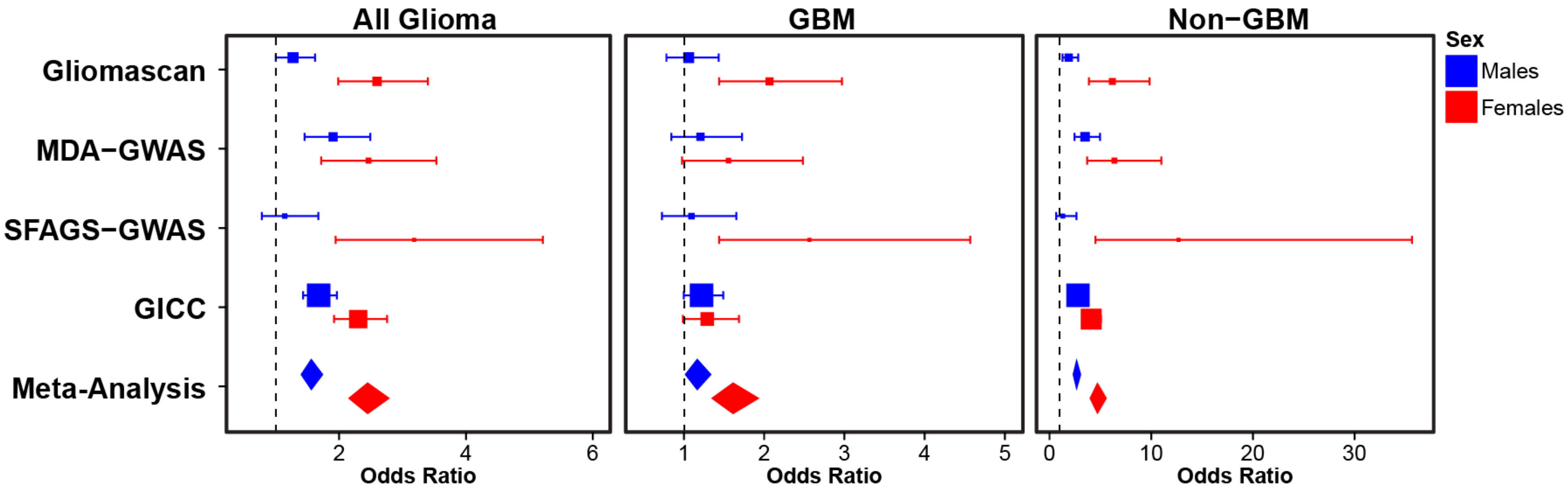

Supplemental Figure 8. Sex-specific odds ratios and 95% CI from meta-analysis and by study for rs9841110 (3p21.31) for all glioma, GBM, and non-GBM

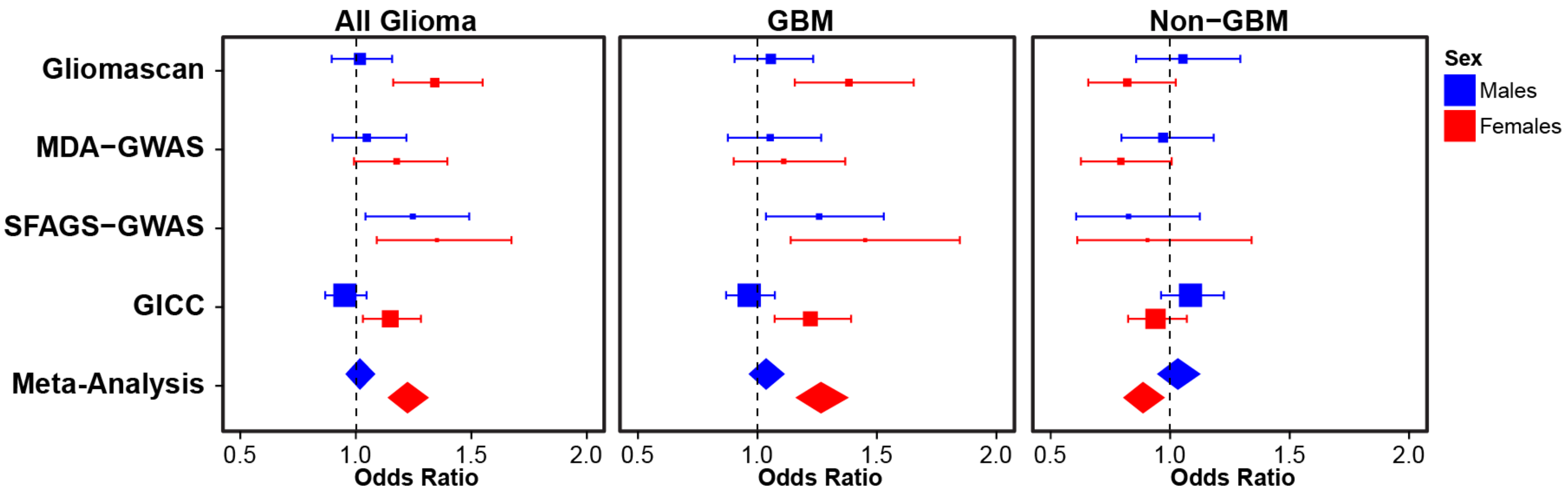

Supplement: Supplementary file 1 — Supplemental Tables 1-7, Supplemental Figures 1-8 [file 41598_2018_24580_MOESM1_ESM.pdf]
